# A simple method to produce *Synechocystis* PCC6803 biofilm under laboratory conditions for electron microscopic and functional studies

**DOI:** 10.1371/journal.pone.0236842

**Published:** 2020-07-30

**Authors:** Ivy Mallick, Prithwiraj Kirtania, Milán Szabó, Faiza Bashir, Ildiko Domonkos, Peter B. Kós, Imre Vass

**Affiliations:** 1 Institute of Plant Biology, Biological Research Centre, Hungarian Academy of Sciences, Szeged, Hungary; 2 Biology PhD School, Faculty of Science and Informatics, University of Szeged, Szeged, Hungary; 3 Department of Biotechnology, Faculty of Science and Informatics, University of Szeged, Szeged, Hungary; University of Hyderabad School of Life Sciences, INDIA

## Abstract

Cyanobacteria can form biofilms in nature, which have ecological roles and high potential for practical applications. In order to study them we need biofilm models that contain healthy cells and can withstand physical manipulations needed for structural studies. At present, combined studies on the structural and physiological features of axenic cyanobacterial biofilms are limited, mostly due to the shortage of suitable model systems. Here, we present a simple method to establish biofilms using the cyanobacterium *Synechocystis* PCC6803 under standard laboratory conditions to be directly used for photosynthetic activity measurements and scanning electron microscopy (SEM). We found that glass microfiber filters (GMF) with somewhat coarse surface features provided a suitable skeleton to form *Synechocystis* PCC6803 biofilms. Being very fragile, untreated GMFs were unable to withstand the processing steps needed for SEM. Therefore, we used polyhydroxybutyrate coating to stabilize the filters. We found that up to five coats resulted in GMF stabilization and made possible to obtain high resolution SEM images of the structure of the surface-attached cells and the extensive exopolysaccharide and pili network, which are essential features of biofilm formation. By using pulse-amplitude modulated variable chlorophyll fluorescence imaging, it was also demonstrated that the biofilms contain photosynthetically active cells. Therefore, the *Synechocystis* PCC6803 biofilms formed on coated GMFs can be used for both structural and functional investigations. The model presented here is easy to replicate and has a potential for high-throughput studies.

## Introduction

Cyanobacteria were responsible for the transformation of an anoxic to oxygenic atmosphere about 2–3 billion years ago [1]. They tend to form biofilms on natural surfaces along with free-living forms in nature [2] and have been found to be one of the influential organisms in shaping up the surrounding environment they thrive in [3, 4].

Cyanobacteria have enormous potential to produce fuels [5, 6], value-added compounds [7, 8] and to be utilized in wastewater treatment [5, 9] in a sustainable way. However, the utilization of this potential needs special arrangement of the cells, in order to ensure optimal light penetration, CO_2_ supply and access to the waste products which should be eliminated. Application of biofilms can be very useful in realizing this potential as in the case of biofilm photobioreactors [10–12].

For any bacterial biofilm formation the first step is the attachment of bacterial cells to a surface followed by gradual morphological changes [13, 14]. The main characteristic of biofilm formation is secretion of exopolysaccharides, which finally form an extensive net-like structure that serves as both protection and source of nutrition for the surviving cells. Since bacteria undergo extensive morphological changes in a biofilm, one of the best ways to study this process is using scanning electron microscopy (SEM) which can reveal the surface structure of the biofilm-forming bacteria in detail, conserving the spatial arrangement on the attached surface [15, 16]. However, so far only few studies have utilized this approach for cyanobacterial biofilms, in a large part due to the lack of efficient methods to produce cyanobacterial biofilms under well-reproducible laboratory conditions.

For laboratory studies it is very useful to use well-characterized model organisms with known genetic background. In this context, characterizing biofilms of a model cyanobacterium named *Synechocystis PCC6803* (*Synechocystis* from now on) would be beneficial, and recent trends in cyanobacterial studies are moving in this direction [17–22]. *Synechocystis* is a freshwater photosynthetic cyanobacterium which was isolated first from a freshwater lake in Oakland, California, USA in 1968 and was identified as a unicellular organism with coccoid cells [23, 24]. Its genome was the first among cyanobacteria to be fully sequenced and annotated in 1996 [24, 25]. Studies on *Synechocystis* have resulted in deep insights into its genetics and further biotechnological manipulation, and it has now been considered as a green *E*. *coli* for plant biologists [26]. It has been developed as a phototrophic cell factory for modelling physiological properties, production of various metabolic products, such as biofuels etc. [27]. As plastids are believed to have evolved from cyanobacteria [25], *Synechocystis* has been the model organism to understand photosynthesis by utilizing its simple genetic background and easily measurable photosynthetic parameters [28, 29]. On this background *Synechocystis* should be a good model organism to understand and manipulate cyanobacterial biofilms. For this reason, there are studies being made using *Synechocystis* biofilms for scientific and technological endeavours using outdoor facilities or special equipment [17, 19–22]; however, the produced biofilms have not been studied by SEM, or if so, well-resolved characteristic structural features of the biofilm have not been observed [30].

Here, we represent for the first time a simple method for developing a photosynthetically active *Synechocystis* biofilm on a glass microfibre filter with standard BG11 medium under normal laboratory conditions, which can be used directly for SEM studies and photosynthetic activity measurements. We believe that this method could yield a suitable model for high-throughput studies on *Synechocystis* biofilms.

## Materials and methods

### Cyanobacterial cultures, growth media and conditions

*Synechocystis* PCC6803 cells were grown in BG11 medium under 40 μmol photons m^-2^ s^-1^ intensity white light, in 3% CO_2_-enriched atmosphere at 30°C. For the experiments cells were adjusted to 5μg/mL chlorophyll-a concentration.

### Biofilm formation on different membranes under laboratory conditions

*Synechocystis* was grown till mid-log phase in liquid cultures and then the cultures were adjusted to 5μg/mL chlorophyll-a concentration. Respective membranes (Millipore Isopore Membrane Filter, and Whatman glass microfibre GF/C filters further called as GMF) were cut into small pieces of about 5mm^2^ area suitable for SEM and sterilized. Finally, the sterilized filter paper pieces were placed in standard-sized Petri dishes filled with 10 mL of sterile BG11 medium. Approximately 10 pieces of filter paper were placed in each dish. On top of each GMF piece 5 μL of *Synechocystis* culture was spotted. The whole system was then kept in an incubator at 30°C for 7 days under 40 μmol photons m^-2^ s^-1^ white light intensity without any disturbance. The membranes were next fixed and studied under SEM to visualize the biofilm. For coating the GMFs, a single granule (approximate weight 2.8 mg) of polyhydroxybutyrate (PHB, from GoodFellow Cambridge Limited) was dissolved in hot chloroform (70°C) and the autoclaved membrane pieces were dipped into it one by one with a forceps and subsequently dried. For multiple coating, membrane pieces were allowed to dry before another dipping for another coat.

### Scanning electron microscopy

The filters covered by biofilms of *Synechocystis* cells were fixed in phosphate buffer (pH 7.4) containing 2.5% glutaraldehyde (Sigma-Aldrich) and 0.15% Alcian blue 8GS (ROTH) for 4 hours. Following post-fixation in 1% OsO_4_ for 1 hour, the samples were dehydrated in aqueous solutions of increasing ethanol concentrations, critical point dried, covered with10 nm gold by a Quorum Q150T ES sputter and observed in a JEOL JSM-7100F/LV scanning electron microscope using 5 kV accelerating voltage under different magnifications.

### Measuring the photosynthetic activity of biofilms grown on GMF

The above mentioned methodology was repeated to form the biofilm on GMF membranes three times coated by PHB. To investigate the photosynthetic efficiency, 4 membrane pieces with *Synechocystis* biofilm were taken out aseptically into a Petri-dish on day seven and photosynthetic activity was measured by using an Pulse-Amplitude Modulation (PAM) fluorescence imaging system (Imaging-PAM M-series Chlorophyll Fluorometer Heinz Walz GmbH, Germany) equipped with a IMAG-MAX/K2 camera and IMAG-MAX/L LED-Array Illumination Unit (Heinz-Walz GmbH, Effeltrich, Germany). The membranes carrying the biofilm were dark-adapted for three minutes before the measurement. The minimum fluorescence in the dark-adapted state (F_o_) was measured by applying a weak measuring light (PPFD <0.3 μmol photons m^-2^ s^-1^), and maximum fluorescence in the dark-adapted state (F_m_) was recorded upon the application of a saturation pulse (with a length of 0.8 s and a PPFD of approx. 2000 μmol photons m^-2^ s^-1^). The maximal quantum yield of Photosystem II (F_v_/F_m_) was calculated as:

$${\text{F}}_{\text{v}}/{\text{F}}_{\text{m}}=\left({\text{F}}_{\text{m}}-{\text{F}}_{\text{o}}\right)/{\text{F}}_{\text{m}}$$ (1)

The relative electron transport rate of PSII (ETR-II) as a function of irradiance was determined using a pre-programmed rapid light curve protocol (using 20 s illumination steps with progressively increasing actinic irradiance). ETR-II was calculated as:

ETR−II=Y(II)*PAR*0.84*0.5 (2)

where Y(II) is the effective quantum yield of PSII at a given irradiance, PAR is the photosynthetically active radiation (in μmol photons m^-2^ s^-1^), the factors 0.84 and 0.5 are the assumed values of absorptivity and the fraction of the absorbed light distributed to PSII, respectively. Non-photochemical quenching (NPQ) was determined using a pre-programmed fluorescence induction-recovery protocol. After determination of F_m_ in darkness, samples were illuminated with actinic light (450 μmol photons m^-2^ s^-1^) for 8 min, during which saturation pulses were given at every 30 s to determine F_m_’, the maximum fluorescence during illumination. Samples were kept in darkness in the recovery phase, during which saturation pulses were given at progressively increasing time intervals to monitor the recovery of F_m_’ (and the relaxation of NPQ) after the illumination period. NPQ was calculated as:

$$\text{NPQ}=\left({\text{F}}_{\text{m}}/{\text{F}}_{\text{m}}’\right)-1$$ (3)

## Results and discussion

As mentioned earlier, complete understanding of cyanobacterial biofilms is at its inception and much needed within the current cyanobacterial research trends. Therefore, this work was initiated to establish a simple, efficient and rapid method to generate *Synechocystis* biofilms under controlled laboratory conditions that can be easily used for SEM and other studies. In order to avoid structural damage of the biofilms during transfer from their growth site to a SEM-compatible membrane, we aimed to grow the biofilm directly on a membrane which is suitable for physiological, SEM and other investigations. We also wanted to take advantage of this approach to investigate whether surface physicochemical properties could affect biofilm formation. To achieve this, we modified some existing protocols that had been used to study biofilms using SEM [31, 32].

**Fig 1:**
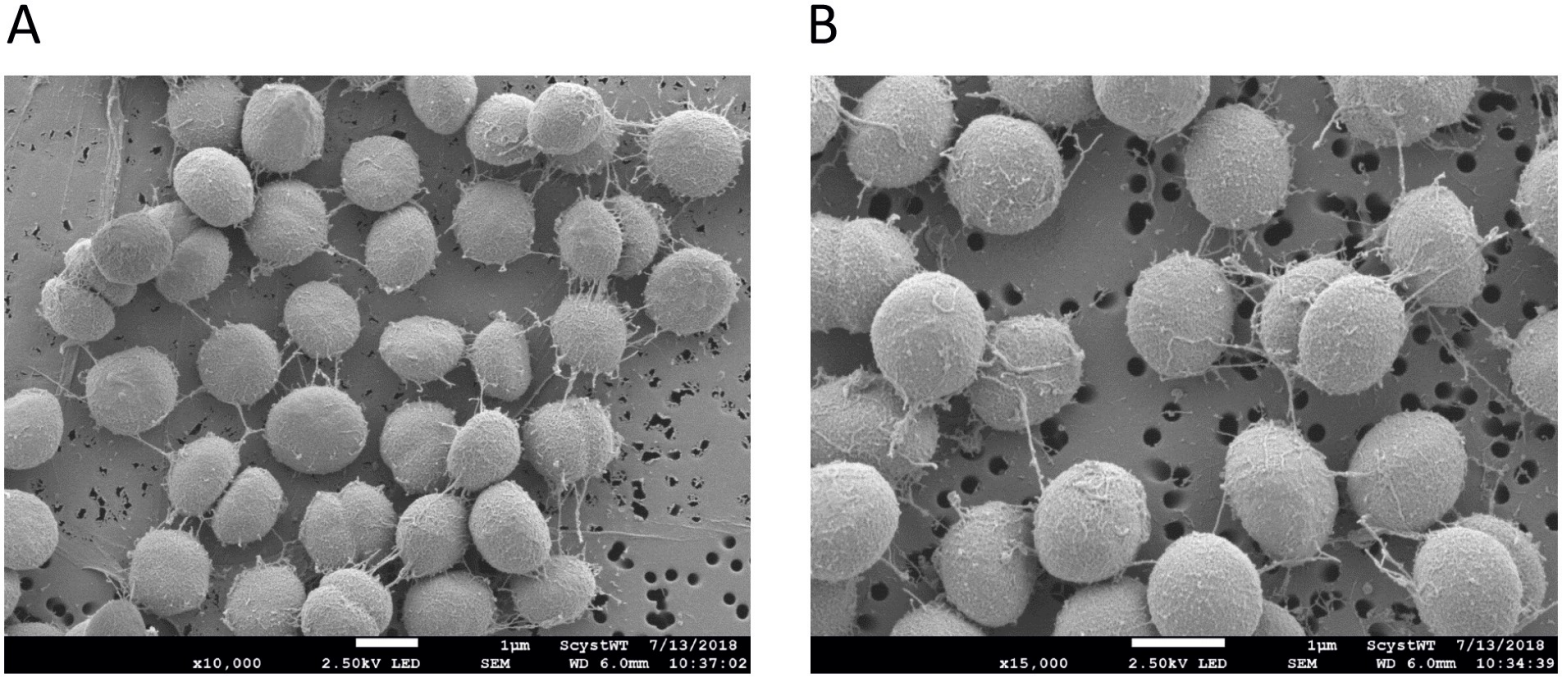
SEM image of *Synechocystis* cells grown on Millipore Isopore Membrane Filter after 7 days of incubation. (A) pili formation at 10000X magnification, (B) pili formation at 15000X magnification (Bar is 1 μm in each panel).

Although it is known that surface physicochemical properties have an influence on bacterial adhesion, so far only a few studies have been carried out to investigate microalgal/cyanobacterial biofilm formation on different materials [33, 34]. In the first step, we aimed to use the Millipore Isopore Membrane Filter with small pore size (0.2μM) for biofilm growth. Small, ca. 5 mm^2^ membrane pieces were autoclaved and then inoculated with *Synechocystis* and kept in a Petri dish under normal growth conditions in an incubator under 3% CO_2_ enriched atmosphere. After seven days, the membranes were subjected to SEM. Fig 1A and 1B (different magnifications) show that the cells remained attached on the membrane even after the process used for SEM fixation. Although pili structures were visible for the attachment to other cells or to the surface, the exopolysaccharide network, which is a crucial feature of biofilm formation, was absent. It was hypothesized that the inability of *Synechocystis* to form a biofilm was related to the improper adherence of cells to the surface used, as suggested by the study of Barros et al., 2019 [33].

**Fig 2:**
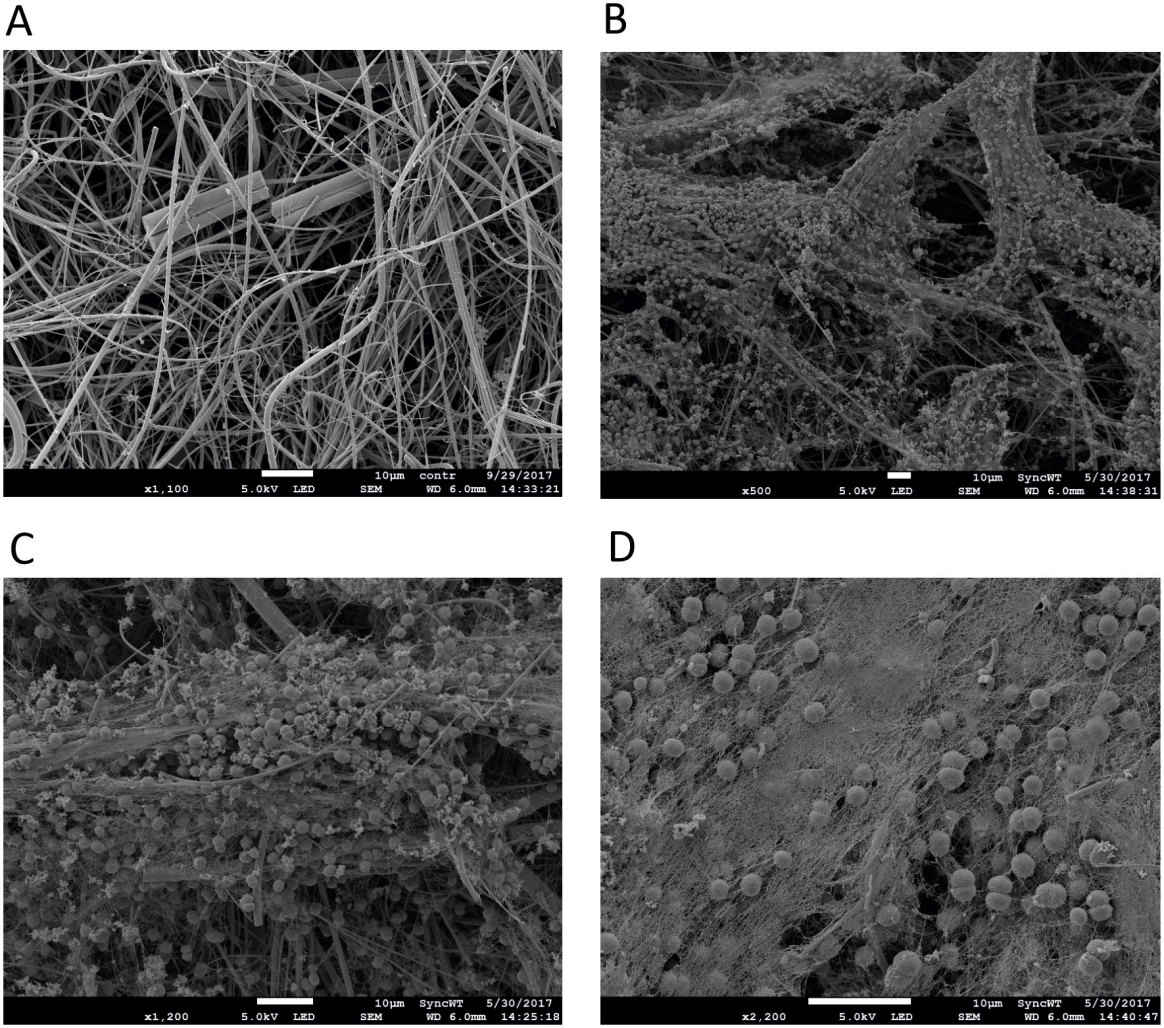
SEM of biofilm formation by *Synechocystis* on glass microfiber filters after 7 days of incubation. (A) control filter at 1100X magnification, (B) biofilm structure formation at 500X magnification, (C) at 1200X magnification, (D) at 2200X magnification (Bar is 10 μm in each panel).

**Fig 3:**
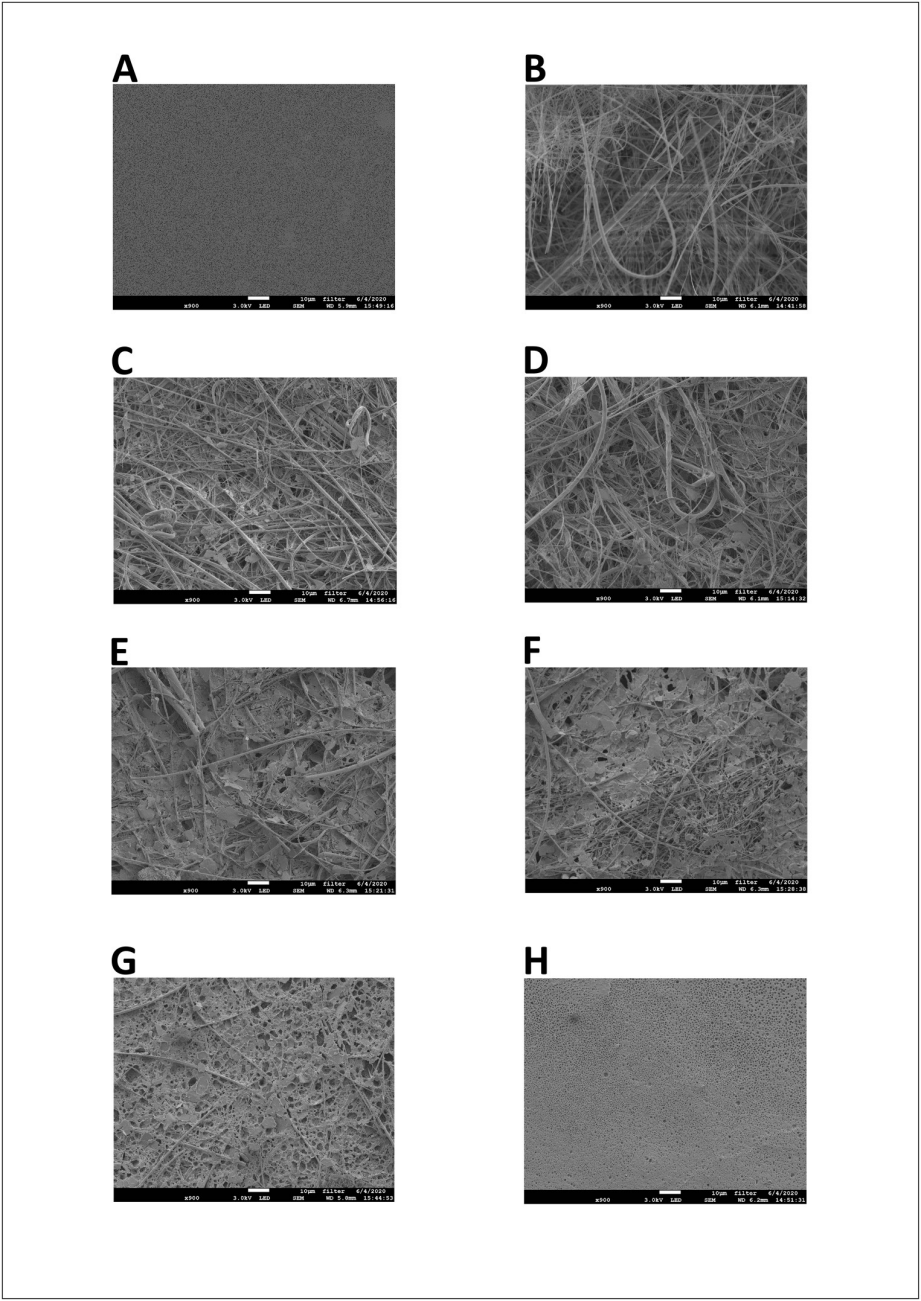
SEM of membrane surfaces coated with various layers of PHB. The Isopore Membrane Filter is shown in the native, uncoated state (A). GMF filters are shown either without coating (B), or with 1 (C), 2 (D), 3 (E), 5 (F), 10 (G) and 15 (H) layers of PHB. Each picture is shown at 900X magnification, with 10 μm bar.

Since we hypothesized that the cause of the failure of biofilm formation might be the smoothness of the surface, which is not suitable for cell adhesion, we wanted to test other membranes with different surface characteristics. Glass microfibre filters (GMFs) were tested for this purpose, as they have a visible roughness. With the method described above, after seven days of incubation we could observe formation of *Synechocystis* biofilms on GMFs that were durable enough for SEM studies. Fig 2B–2D show that *Synechocystis* forms a biofilm on the GMF membrane, since the electron micrographs display a well-built exopolysaccharide network along with pili among the cells. The figure shows images with different magnifications to demonstrate the mesh-like structures around the *Synechocystis* cells formed due to exopolysaccharide secretion. Although the roughness of the GMF filter surface provided proper support to adhering *Synechocystis* cells, the stability of the GMFs was a matter of concern for the whole process. It is evident from the images (Figs 2A and 3B) that the GMF membrane was very fragile and it could not withstand harsh treatments during sample preparation for SEM, leading to damage of the biofilm structures.

The objective of the next step was to find ways to stabilize the GMF membrane so that it could withstand the treatments for SEM sample preparation and still provide sufficiently rough surface for adequate cell attachment. Therefore, we used a stabilizing coating on the membrane to increase the integrity of the surface. Polyhydroxybutyrate (PHB) is a well-known biopolymer which can form a water-resistant coating on different surfaces with a biocompatible nature [35]. Importantly, *Synechocystis* can synthesize and accumulate PHB globules intracellularly as a carbon and energy storage compound when grown under nitrogen- and phosphorus-starved conditions [36–38], which proves its biocompatibility. Therefore PHB coating appeared to be a safe option for stabilizing membranes, considering the natural affinity of *Synechocystis* for PHB.

For this reason, we coated the sterilized GMF membranes with one to fifteen PHB layers aseptically, which were then used to test *Synechocystis* biofilm formation. The electron micrographs show that coating of GMFs with PHB preserved membrane stability during the sample preparation step for SEM, but also resulted in an increasing extent of smoothness, especially above 10 coating layers (Fig 3C–3H for 1, 2, 3, 5, 10 and 15 layers, respectively). For comparison, the uncoated Isopore Membrane Filter is shown (Fig 3A), which had the highest level of smoothness.

**Fig 4:**
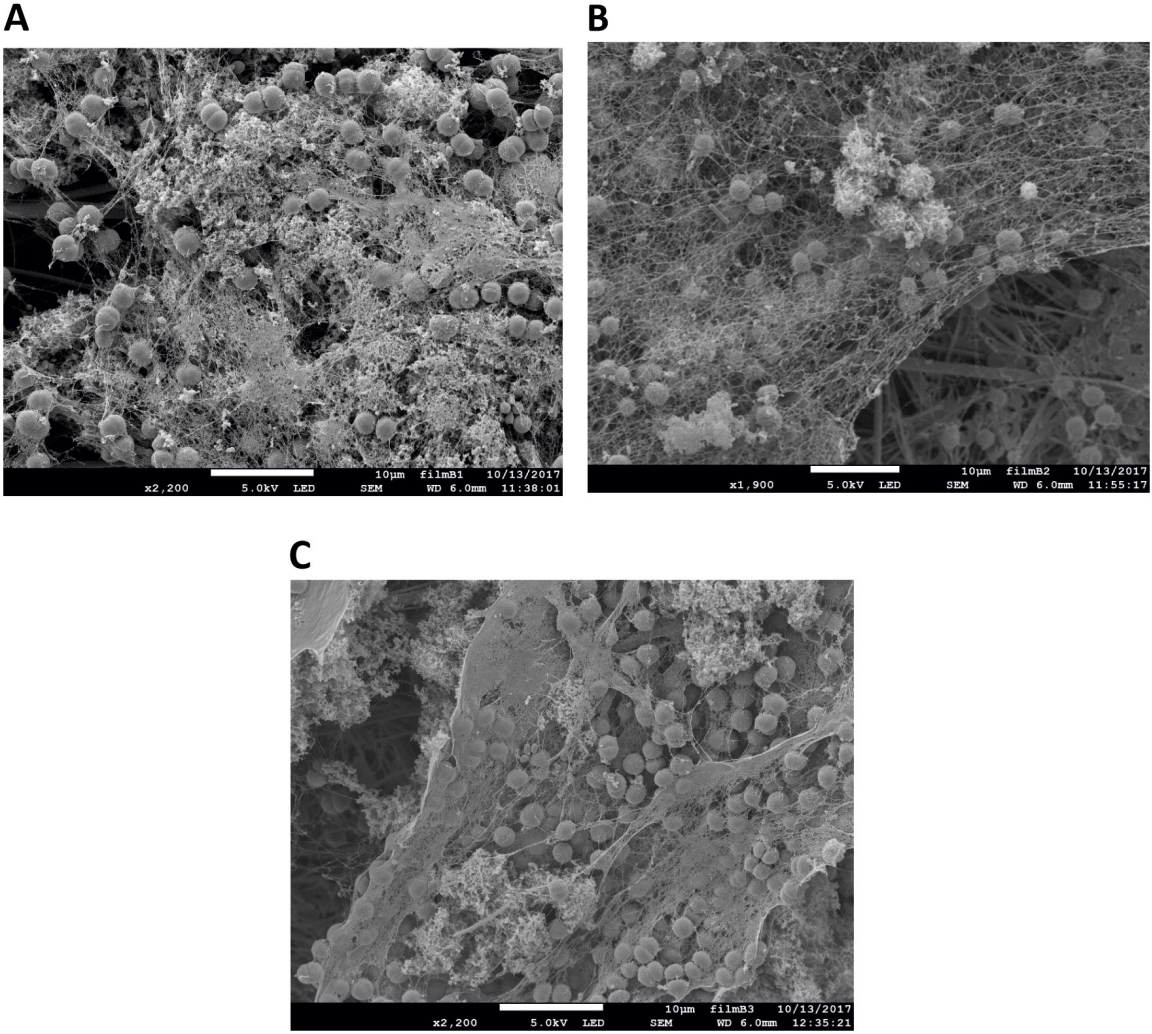
SEM of biofilm formation by *Synechocystis* on glass microfiber filter coated with PHB after 7 days of incubation. (A) 1 layer at 2200X magnification, (B) 2 layers at 1900X magnification, (C) 3 layers of PHB coating at 2200X magnification (Bar is 10 μm in each panel).

Incubation of 1, 2 and 3 times PHB coated GMF membranes with *Synechocystis* cells demonstrated biofilm formation with extensive exopolysaccharide and pili network (Fig 4).

**Fig 5:**
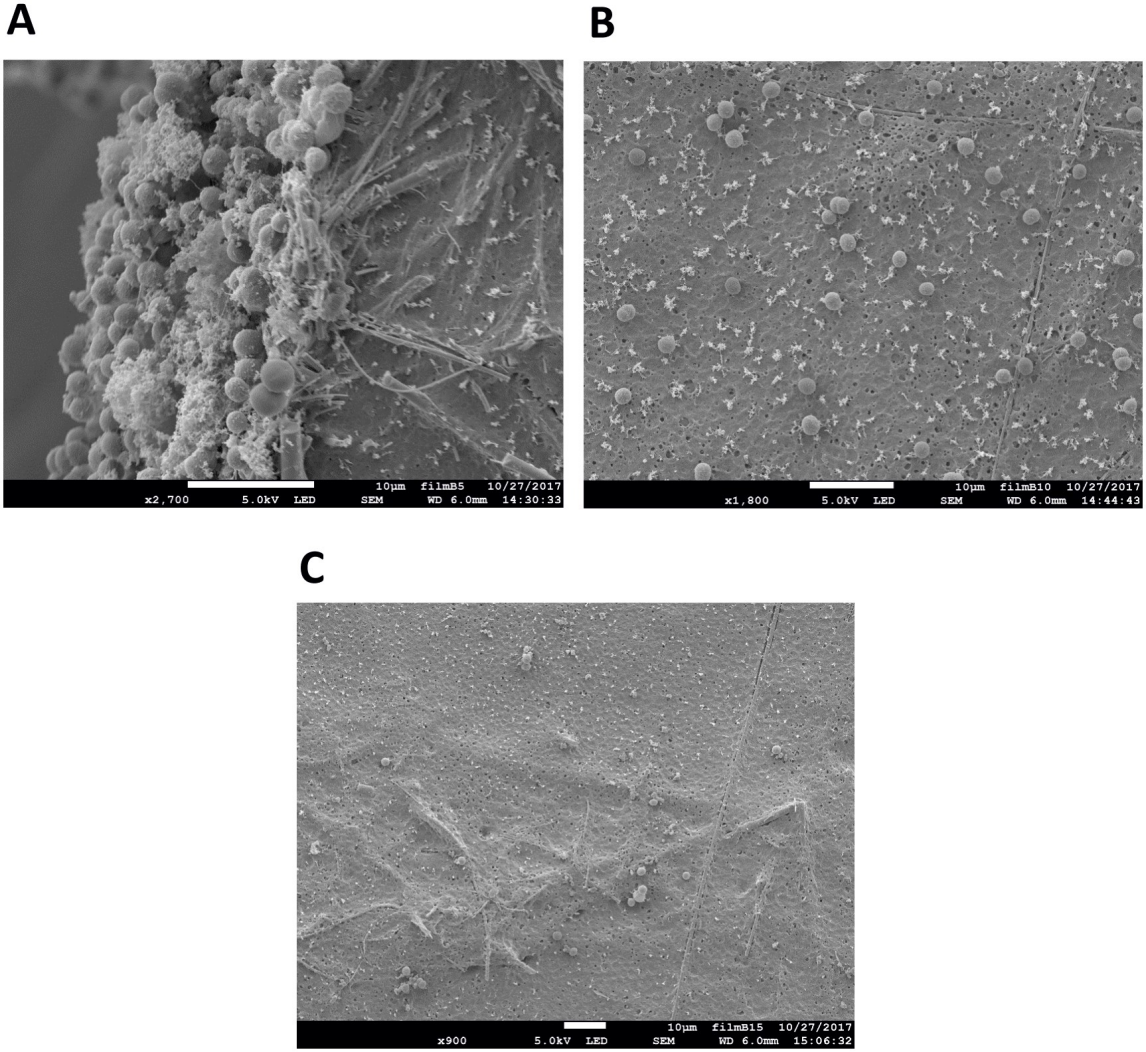
SEM of biofilm formation by *Synechocystis* on glass microfiber filter coated with PHB after 7 days of incubation. (A) 5 layers at 2700X magnification, (B) 10 layers at 1800X magnification, (C) 15 layers of PHB coating at 900X magnification (Bar is 10 μm in each panel).

Based on the above results, we wanted to check if further improvement of biofilm formation could be made by increasing the number of coating layers, which increases the stability of the surface but at the same time increases its smoothness as well (Fig 3F–3H). The electron micrographs in Fig 5 show that when the GMFs were coated with up to five layers of PHB, *Synechocystis* was able to form biofilms (Fig 5A). However, when ten (Fig 5B) or 15 layers (Fig 5C) were used, there was no visible biofilm formation, as the cells could not attach to the film surfaces. For Fig 5B and 5C, magnifications lower than that in Fig 5A were used in order to demonstrate that most of the surface was empty, lacking any attached cells or biofilm layer. We concluded that, due to excessive coating by PHB, the surface of GMFs were much more than adequately smoothened, resulting in suboptimal attachment of *Synechocystis* for biofilm formation.

**Fig 6:**
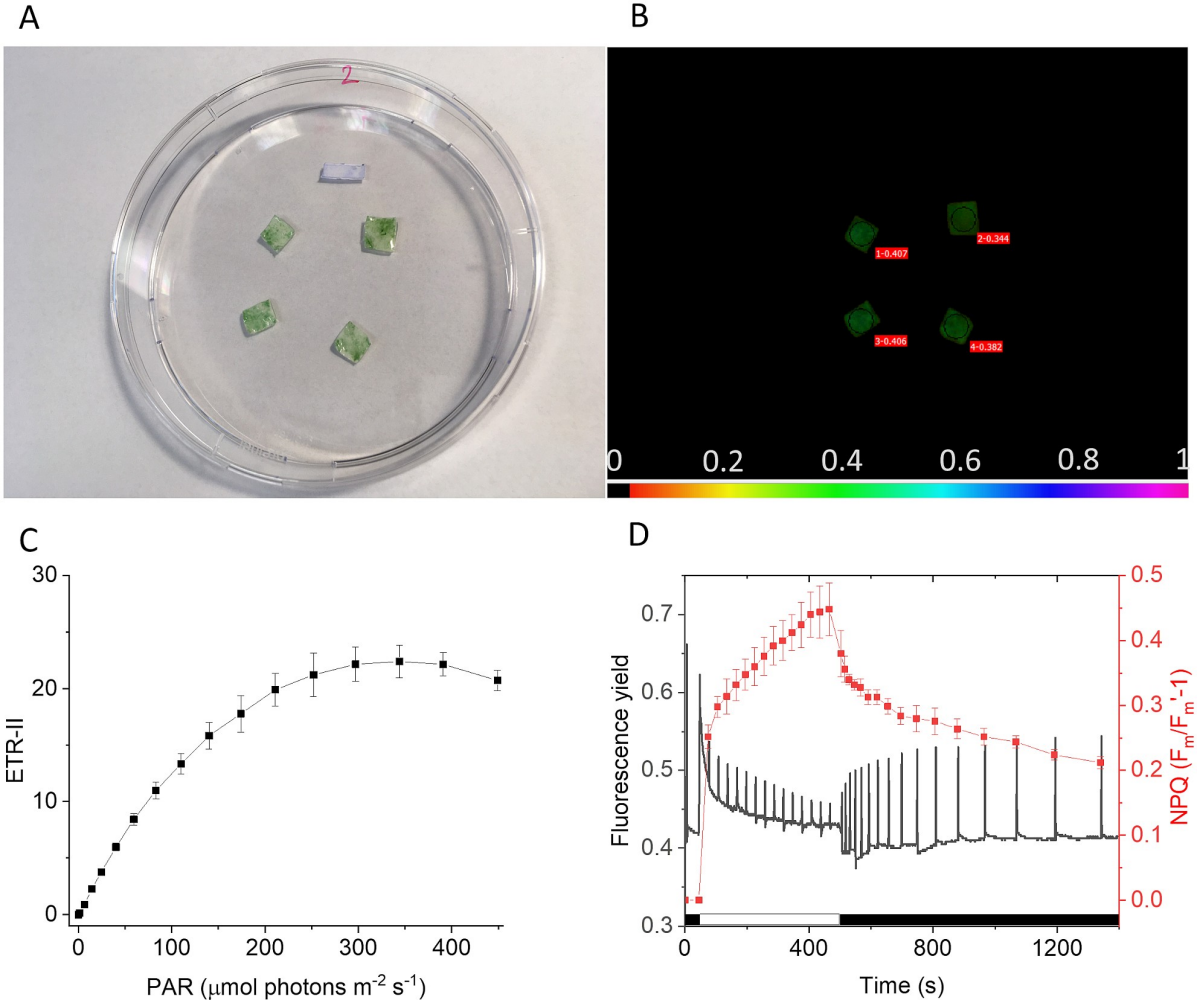
Pulse-Amplitude Modulation (PAM) chlorophyll fluorescence imaging of the GMF carrying *Synechocystis* biofilm. (A) Colour photograph of the *Synechocystis* biofilms grown on 3X PHB coated GMF membranes in a Petri dish (representing one biological experiment with four technical replicates, which were subjected to PAM measurement). The white bar represents 1 cm length. (B) False-colored chlorophyll fluorescence image showing the maximum quantum yield of PSII (F_v_/F_m_) values in the selected areas of interest (black circles). The color bar in panel B represents the color coding of F_v_/F_m_ values (black: F_v_/F_m_ = 0, magenta: F_v_/F_m_ = 1). Measurements of F_v_/F_m_ were performed on two independent biological replicates, each representing four biofilms as technical replicates. (C) Electron transport rate through Photosystem II (ETR-II) vs. irradiance curves of the *Synechocystis* biofilms (n = 4, mean±S.D.), (D) representative fluorescence induction-recovery curves of the *Synechocystis* biofilms (black trace) and the calculated non-photochemical quenching (NPQ, red traces, (n = 4, mean±S.D.). Black bars represent the dark phases, white bar represents the illumination phase using actinic light (450 μmol photons m^-2^ s^-1^ photon flux density).

Natural cyanobacterial biofilms are important communities of living cells [3, 4], therefore it was important to verify if the *Synechocystis* cells attached to the surface of the coated GMFs are photosynthetically active. Chlorophyll fluorescence imaging is a powerful tool to investigate spatial heterogeneity of photosynthetic performance non-invasively on plant leaves and biofilms of cyanobacteria and microalgae; moreover, it is also applicable for screening procedures to identify altered regulation of photosynthesis in the generated mutants [39–41]. For this reason, we used variable chlorophyll fluorescence imaging of PHB-coated membranes, which were incubated with a *Synechocystis* culture for seven days to form a biofilm (Fig 6A). A total of eight membranes were imaged simultaneously (four technical replicates in two biological replicates). The F_v_/F_m_ values were measured on the selected areas of interest from the membranes (black circles in Fig 6B). The results obtained showed F_v_/F_m_ values in the range of 0.344–0.41 for the biofilm layers, with an average of 0.385 ± 0.024 (n = 8) (representative F_v_/F_m_ images of the biofilms are shown in Fig 6). The F_v_/F_m_ values obtained correspond well with the reported range of 0.3–0.4, which is characteristic for liquid *Synechocystis* cultures [42, 43]. This result shows that the biofilms formed on the GMF membranes are photosynthetically active, and therefore have a potential to be used for studying the photosynthetic properties of *Synechocystis* using this model. In order to show the potential of GMF supported biofilms for photosynthesis studies the light intensity dependence of the electron transport rate through Photosystem II (ETR-II) was also measured (Fig 6C). In addition, the widely used fluorescence quenching analysis was also performed, and the non-photochemical quenching parameter (NPQ) was determined during dark-light-dark transitions (Fig 6D) [40, 41].

## Conclusion and future perspectives

The importance of this study is that it presents for the first time an easy and fast method to establish *Synechocystis* biofilm models under standard laboratory conditions that can be used for both physiological and SEM investigations. Besides providing well-resolved details of the exopolysaccharide and pili network in the biofilm by SEM, our model system also made possible to measure various parameters of photosynthetic activity by Pulse-Amplitude Modulation (PAM) chlorophyll fluorescence imaging. The advantage of our method is that it is not dependent on any special equipment, and utilizes simple GMF filters that are readily available in most laboratories. Furthermore, this method has the potential to be adapted for studying multiple mutants of *Synechocystis* and various surface modifications in a high-throughput manner using multiple chambered plates instead of a single Petri dish. By utilizing this approach, a better understanding of biofilm formation as well as optimization of biofilm growth can be achieved for various biotechnological applications in future studies.

## Supporting information

S1 File (PDF)

## Abbreviations

Chl.: Chlorophyll
GMF: Whatman glass microfibre GF/C filters
PHB: polyhydroxybutyrate
SEM: scanning electron microscopy
