# Supplementary material for: A simple method to produce Synechocystis PCC6803 biofilm under laboratory conditions for electron microscopic and functional studies
Source: PLoS One. 2020 Jul 30;15(7):e0236842. doi: 10.1371/journal.pone.0236842 (PMC7392257; doi:10.1371/journal.pone.0236842)
Supplement: S1 File — (PDF) [file pone.0236842.s001.pdf]

Supporting information

A simple method to produce *Synechocystis* PCC6803 biofilm under laboratory conditions for electron microscopic and functional studies

Running title: Biofilm formation by *Synechocystis* PCC6803 on glass microfiber filters.

[1a]Ivy Mallick, [1a]Prithwiraj Kirtania, [1]Milán Szabó, [1,2]Faiza Bashir, [1]Ildiko Domonkos, [1,3]Peter B. Kós, [1*]Imre Vass

[1]Institute of Plant Biology, Biological Research Centre, Hungarian Academy of Sciences, Szeged, Hungary

[2]Biology PhD School, Faculty of Science and Informatics, University of Szeged, Szeged, Hungary

[3]Department of Biotechnology, Faculty of Science and Informatics, University of Szeged, Szeged, Hungary

[a]authors contributed equally

Original chlorophyll fluorescence parameters and the calculated maximum quantum yield of PSII in dark-adapted Synechocystis biofilms (the mean values and standard deviations are presented in the text).

<table>
<tr><th></th><th>Fo1</th><th>Fo2</th><th>Fo3</th><th>Fo4</th><th>Fm1</th><th>Fm2</th><th>Fm3</th><th>Fm4</th><th>Fv/Fm1</th><th>Fv/Fm2</th><th>Fv/Fm3</th><th>Fv/Fm4</th><th>Fv/Fm_mean</th><th>S.D.</th></tr>
<tr><td>replicate 1</td><td>0.068</td><td>0.078</td><td>0.081</td><td>0.065</td><td>0.107</td><td>0.133</td><td>0.129</td><td>0.106</td><td>0.362</td><td>0.41</td><td>0.371</td><td>0.387</td><td rowspan="2">0.384</td><td rowspan="2">0.024</td></tr>
<tr><td>replicate 2</td><td>0.078</td><td>0.072</td><td>0.079</td><td>0.078</td><td>0.131</td><td>0.110</td><td>0.133</td><td>0.127</td><td>0.407</td><td>0.344</td><td>0.406</td><td>0.382</td></tr>
</table>

Original Light Curve data of the Synechocystis biofilms (mean values and standard deviation are plotted in Fig. 6C)

PAR	F1	F2	F3	F4	Fm'1	Fm'2	Fm'3	Fm'4	Y(II)1	Y(II)2	Y(II)3	Y(II)4	ETR1	ETR2	ETR3	ETR4	ETR_mean	S.D.
0	0.078	0.071	0.079	0.078	0.130	0.107	0.130	0.125	0.400	0.338	0.392	0.374	0.00	0.00	0.00	0.00	0.00	0.000
1	0.081	0.076	0.083	0.082	0.129	0.107	0.130	0.124	0.371	0.294	0.362	0.337	0.16	0.12	0.15	0.14	0.14	0.014
6.4	0.086	0.079	0.088	0.086	0.133	0.115	0.134	0.128	0.357	0.312	0.344	0.332	0.96	0.84	0.92	0.89	0.90	0.051
14.5	0.081	0.076	0.083	0.082	0.134	0.115	0.135	0.130	0.392	0.340	0.385	0.37	2.39	2.07	2.34	2.25	2.26	0.141
24.5	0.081	0.076	0.084	0.082	0.133	0.114	0.134	0.128	0.387	0.336	0.376	0.359	3.98	3.46	3.87	3.69	3.75	0.229
40.3	0.082	0.076	0.085	0.082	0.131	0.112	0.132	0.127	0.375	0.325	0.359	0.351	6.35	5.50	6.08	5.94	5.97	0.353
59.3	0.082	0.075	0.084	0.082	0.128	0.109	0.130	0.124	0.354	0.311	0.351	0.336	8.82	7.75	8.74	8.37	8.42	0.489
83.1	0.082	0.076	0.085	0.082	0.123	0.105	0.127	0.120	0.331	0.284	0.329	0.314	11.55	9.91	11.48	10.96	10.98	0.757
110	0.083	0.076	0.086	0.082	0.119	0.102	0.123	0.116	0.302	0.260	0.303	0.291	13.95	12.01	14.00	13.44	13.35	0.928
140	0.083	0.075	0.086	0.083	0.116	0.099	0.120	0.113	0.284	0.243	0.286	0.265	16.70	14.29	16.82	15.58	15.85	1.178
174	0.083	0.075	0.086	0.084	0.112	0.096	0.117	0.110	0.258	0.215	0.264	0.237	18.85	15.71	19.29	17.32	17.79	1.626
211	0.084	0.075	0.088	0.084	0.110	0.094	0.115	0.108	0.236	0.203	0.238	0.222	20.91	17.99	21.09	19.67	19.92	1.431
252	0.085	0.076	0.089	0.085	0.108	0.092	0.113	0.106	0.213	0.176	0.216	0.198	22.54	18.63	22.86	20.96	21.25	1.935
297	0.087	0.076	0.090	0.086	0.106	0.091	0.111	0.104	0.184	0.162	0.190	0.175	22.95	20.21	23.70	21.83	22.17	1.519
344	0.088	0.077	0.091	0.087	0.105	0.090	0.109	0.103	0.164	0.142	0.162	0.153	23.69	20.52	23.41	22.11	22.43	1.451
391	0.089	0.077	0.092	0.087	0.103	0.088	0.107	0.101	0.138	0.128	0.142	0.132	22.66	21.02	23.32	21.68	22.17	1.021
449	0.090	0.078	0.093	0.088	0.101	0.087	0.105	0.099	0.107	0.107	0.117	0.109	20.18	20.18	22.06	20.56	20.74	0.898

Original chlorophyll fluorescence induction-recovery data exported by the ImagingWin v2.41a software of the Imaging-PAM M-series Chlorophyll Fluorometer (Heinz Walz GmbH, Germany), the values are plotted in Fig. 6 D.

Time	F1	F2	F3	F4
0.5	0	0	0	0
1	0.383	0.347	0.405	0.388
1.5	0.383	0.349	0.408	0.391
2	0.383	0.349	0.408	0.391
2.5	0.383	0.349	0.408	0.391
3	0.383	0.349	0.408	0.391
3.5	0.383	0.349	0.408	0.391
4	0.383	0.349	0.408	0.391
4.5	0.383	0.349	0.408	0.391
5	0.383	0.349	0.408	0.391
5.5	0.383	0.349	0.408	0.391
6	0.5	0.432	0.535	0.505
6.5	0.618	0.515	0.662	0.623
7	0.383	0.349	0.408	0.391
7.5	0.393	0.356	0.417	0.393
8	0.405	0.366	0.43	0.398
8.5	0.403	0.366	0.427	0.398
9	0.403	0.366	0.427	0.398
9.5	0.403	0.366	0.427	0.398
10	0.403	0.366	0.427	0.398
10.5	0.403	0.366	0.427	0.398
11	0.403	0.366	0.427	0.398
11.5	0.403	0.366	0.427	0.398
12	0.403	0.366	0.427	0.398
12.5	0.403	0.366	0.425	0.396
13	0.403	0.366	0.425	0.396
13.5	0.403	0.366	0.427	0.398
14	0.403	0.366	0.427	0.398
14.5	0.403	0.364	0.425	0.398

15	0.403	0.364	0.425	0.398
15.5	0.403	0.366	0.425	0.396
16	0.403	0.366	0.425	0.396
16.5	0.403	0.364	0.425	0.396
17	0.403	0.364	0.425	0.396
17.5	0.4	0.364	0.425	0.398
18	0.4	0.364	0.425	0.398
18.5	0.4	0.364	0.422	0.398
19	0.4	0.364	0.422	0.398
19.5	0.4	0.364	0.422	0.398
20	0.4	0.364	0.422	0.398
20.5	0.4	0.364	0.425	0.398
21	0.4	0.364	0.425	0.398
21.5	0.4	0.364	0.422	0.398
22	0.4	0.364	0.422	0.398
22.5	0.398	0.364	0.422	0.398
23	0.398	0.364	0.422	0.398
23.5	0.398	0.364	0.422	0.398
24	0.398	0.364	0.422	0.398
24.5	0.398	0.361	0.422	0.398
25	0.398	0.361	0.422	0.398
25.5	0.398	0.361	0.422	0.398
26	0.398	0.361	0.422	0.398
26.5	0.398	0.361	0.422	0.398
27	0.398	0.361	0.42	0.398
27.5	0.398	0.361	0.42	0.398
28	0.398	0.361	0.422	0.398
28.5	0.398	0.361	0.422	0.398
29	0.398	0.361	0.42	0.398
29.5	0.398	0.361	0.422	0.398
30	0.398	0.361	0.42	0.398
30.5	0.398	0.361	0.42	0.398
31	0.398	0.361	0.42	0.398
31.5	0.398	0.361	0.42	0.398

32	0.398	0.359	0.42	0.398
32.5	0.398	0.359	0.42	0.398
33	0.396	0.361	0.42	0.398
33.5	0.396	0.361	0.42	0.398
34	0.398	0.359	0.42	0.398
34.5	0.398	0.359	0.42	0.398
35	0.396	0.359	0.42	0.398
35.5	0.396	0.359	0.42	0.398
36	0.396	0.359	0.42	0.398
36.5	0.396	0.359	0.42	0.398
37	0.396	0.359	0.42	0.398
37.5	0.396	0.359	0.42	0.398
38	0.396	0.359	0.42	0.398
38.5	0.396	0.359	0.42	0.398
39	0.396	0.359	0.42	0.398
39.5	0.396	0.359	0.42	0.398
40	0.396	0.359	0.42	0.398
40.5	0.396	0.359	0.42	0.398
41	0.396	0.359	0.42	0.398
41.5	0.396	0.359	0.42	0.398
42	0.396	0.359	0.42	0.398
42.5	0.396	0.359	0.42	0.398
43	0.396	0.359	0.417	0.398
43.5	0.396	0.359	0.417	0.398
44	0.396	0.359	0.42	0.398
44.5	0.396	0.359	0.42	0.398
45	0.396	0.361	0.42	0.403
45.5	0.425	0.369	0.449	0.427
46	0.444	0.378	0.471	0.444
46.5	0.461	0.383	0.488	0.461
47	0.5	0.415	0.532	0.503
47.5	0.542	0.449	0.576	0.544
48	0.583	0.483	0.623	0.586
48.5	0.571	0.454	0.598	0.562

49	0.557	0.425	0.574	0.537
49.5	0.554	0.425	0.574	0.542
50	0.552	0.422	0.571	0.54
50.5	0.547	0.42	0.569	0.535
51	0.544	0.417	0.564	0.532
51.5	0.54	0.415	0.562	0.53
52	0.537	0.415	0.562	0.53
52.5	0.535	0.413	0.557	0.525
53	0.53	0.408	0.552	0.522
53.5	0.527	0.408	0.547	0.518
54	0.522	0.405	0.547	0.518
54.5	0.52	0.405	0.544	0.515
55	0.518	0.403	0.54	0.513
55.5	0.513	0.4	0.535	0.508
56	0.508	0.398	0.535	0.505
56.5	0.508	0.398	0.532	0.503
57	0.505	0.398	0.532	0.503
57.5	0.5	0.396	0.53	0.5
58	0.498	0.396	0.527	0.498
58.5	0.493	0.393	0.525	0.496
59	0.493	0.391	0.522	0.493
59.5	0.493	0.391	0.522	0.491
60	0.488	0.388	0.52	0.491
60.5	0.486	0.388	0.52	0.488
61	0.483	0.386	0.518	0.486
61.5	0.483	0.388	0.518	0.486
62	0.483	0.388	0.515	0.486
62.5	0.481	0.386	0.513	0.483
63	0.476	0.386	0.51	0.481
63.5	0.476	0.386	0.51	0.479
64	0.476	0.386	0.51	0.479
64.5	0.474	0.386	0.51	0.479
65	0.471	0.383	0.508	0.474
65.5	0.469	0.383	0.505	0.474

66	0.469	0.383	0.503	0.474
66.5	0.469	0.383	0.503	0.474
67	0.466	0.383	0.503	0.471
67.5	0.464	0.381	0.5	0.471
68	0.464	0.381	0.5	0.469
68.5	0.461	0.378	0.5	0.469
69	0.464	0.381	0.5	0.469
69.5	0.461	0.381	0.498	0.466
70	0.461	0.378	0.498	0.466
70.5	0.459	0.378	0.498	0.466
71	0.459	0.378	0.498	0.466
71.5	0.459	0.378	0.498	0.466
72	0.457	0.378	0.498	0.466
72.5	0.457	0.378	0.496	0.464
73	0.454	0.378	0.493	0.461
73.5	0.454	0.378	0.491	0.461
74	0.454	0.378	0.491	0.461
74.5	0.452	0.376	0.491	0.459
75	0.452	0.376	0.488	0.459
75.5	0.449	0.376	0.486	0.457
76	0.449	0.376	0.486	0.457
76.5	0.452	0.376	0.486	0.457
77	0.464	0.388	0.503	0.469
77.5	0.476	0.403	0.52	0.481
78	0.488	0.417	0.537	0.493
78.5	0.469	0.393	0.508	0.461
79	0.447	0.366	0.479	0.43
79.5	0.447	0.369	0.479	0.432
80	0.447	0.369	0.479	0.435
80.5	0.447	0.369	0.479	0.435
81	0.442	0.369	0.476	0.437
81.5	0.442	0.369	0.474	0.435
82	0.442	0.369	0.476	0.437
82.5	0.442	0.369	0.476	0.439

83	0.442	0.371	0.476	0.439
83.5	0.439	0.369	0.474	0.437
84	0.437	0.369	0.474	0.437
84.5	0.439	0.369	0.474	0.439
85	0.439	0.371	0.474	0.442
85.5	0.439	0.371	0.474	0.439
86	0.437	0.371	0.471	0.439
86.5	0.437	0.369	0.471	0.439
87	0.439	0.371	0.471	0.439
87.5	0.439	0.371	0.474	0.442
88	0.437	0.371	0.471	0.442
88.5	0.437	0.369	0.471	0.439
89	0.435	0.369	0.469	0.439
89.5	0.435	0.371	0.471	0.439
90	0.437	0.371	0.471	0.442
90.5	0.435	0.371	0.471	0.439
91	0.432	0.369	0.469	0.437
91.5	0.432	0.369	0.469	0.437
92	0.432	0.369	0.469	0.437
92.5	0.432	0.369	0.469	0.439
93	0.435	0.371	0.469	0.437
93.5	0.432	0.369	0.466	0.437
94	0.432	0.369	0.466	0.437
94.5	0.432	0.369	0.466	0.437
95	0.432	0.369	0.469	0.437
95.5	0.432	0.369	0.466	0.437
96	0.43	0.366	0.466	0.435
96.5	0.43	0.369	0.466	0.437
97	0.432	0.369	0.466	0.437
97.5	0.432	0.369	0.466	0.437
98	0.43	0.369	0.466	0.435
98.5	0.43	0.366	0.466	0.435
99	0.427	0.366	0.464	0.435
99.5	0.427	0.366	0.464	0.437

100	0.427	0.366	0.466	0.437
100.5	0.427	0.366	0.464	0.435
101	0.427	0.364	0.464	0.432
101.5	0.427	0.364	0.464	0.432
102	0.427	0.364	0.464	0.435
102.5	0.427	0.364	0.464	0.435
103	0.427	0.364	0.464	0.432
103.5	0.425	0.364	0.461	0.432
104	0.425	0.364	0.464	0.432
104.5	0.425	0.364	0.464	0.432
105	0.425	0.366	0.464	0.435
105.5	0.425	0.364	0.464	0.432
106	0.425	0.364	0.464	0.432
106.5	0.425	0.364	0.464	0.432
107	0.439	0.376	0.481	0.444
107.5	0.454	0.388	0.498	0.459
108	0.469	0.403	0.518	0.474
108.5	0.447	0.381	0.493	0.452
109	0.425	0.359	0.466	0.427
109.5	0.425	0.359	0.466	0.427
110	0.425	0.359	0.466	0.425
110.5	0.425	0.361	0.464	0.427
111	0.422	0.359	0.464	0.427
111.5	0.422	0.361	0.464	0.427
112	0.422	0.361	0.464	0.43
112.5	0.422	0.361	0.461	0.427
113	0.422	0.361	0.461	0.427
113.5	0.422	0.361	0.461	0.43
114	0.422	0.361	0.461	0.43
114.5	0.422	0.361	0.461	0.427
115	0.422	0.364	0.461	0.427
115.5	0.42	0.364	0.461	0.427
116	0.42	0.361	0.459	0.427
116.5	0.42	0.364	0.459	0.427

117	0.422	0.364	0.459	0.427
117.5	0.42	0.364	0.459	0.427
118	0.42	0.361	0.459	0.427
118.5	0.42	0.364	0.459	0.427
119	0.422	0.364	0.459	0.427
119.5	0.42	0.364	0.459	0.427
120	0.42	0.364	0.457	0.427
120.5	0.417	0.361	0.459	0.425
121	0.42	0.361	0.459	0.427
121.5	0.42	0.364	0.459	0.427
122	0.42	0.364	0.459	0.427
122.5	0.417	0.361	0.459	0.427
123	0.417	0.361	0.457	0.427
123.5	0.417	0.361	0.459	0.427
124	0.417	0.361	0.459	0.43
124.5	0.417	0.361	0.459	0.427
125	0.417	0.361	0.457	0.427
125.5	0.417	0.361	0.457	0.427
126	0.417	0.361	0.457	0.427
126.5	0.417	0.364	0.457	0.427
127	0.417	0.361	0.457	0.427
127.5	0.415	0.361	0.454	0.425
128	0.415	0.361	0.454	0.425
128.5	0.415	0.361	0.454	0.425
129	0.415	0.361	0.454	0.425
129.5	0.415	0.361	0.454	0.425
130	0.415	0.361	0.454	0.425
130.5	0.415	0.361	0.454	0.422
131	0.415	0.361	0.454	0.425
131.5	0.415	0.361	0.454	0.427
132	0.415	0.361	0.454	0.425
132.5	0.415	0.361	0.454	0.425
133	0.415	0.359	0.454	0.425
133.5	0.415	0.359	0.457	0.425

134	0.415	0.361	0.457	0.425
134.5	0.415	0.359	0.454	0.425
135	0.415	0.361	0.454	0.425
135.5	0.415	0.359	0.454	0.425
136	0.415	0.361	0.454	0.425
136.5	0.417	0.361	0.457	0.427
137	0.43	0.374	0.474	0.439
137.5	0.444	0.386	0.491	0.452
138	0.459	0.4	0.508	0.466
138.5	0.437	0.386	0.481	0.447
139	0.415	0.369	0.452	0.425
139.5	0.415	0.369	0.452	0.427
140	0.415	0.369	0.452	0.427
140.5	0.413	0.366	0.452	0.427
141	0.413	0.364	0.452	0.427
141.5	0.415	0.364	0.452	0.427
142	0.415	0.366	0.454	0.427
142.5	0.415	0.364	0.454	0.425
143	0.415	0.364	0.452	0.425
143.5	0.413	0.364	0.452	0.422
144	0.415	0.361	0.452	0.425
144.5	0.415	0.364	0.454	0.425
145	0.415	0.361	0.452	0.422
145.5	0.413	0.361	0.452	0.42
146	0.413	0.361	0.452	0.422
146.5	0.413	0.361	0.454	0.422
147	0.415	0.361	0.452	0.422
147.5	0.41	0.361	0.452	0.42
148	0.413	0.361	0.449	0.422
148.5	0.41	0.359	0.452	0.422
149	0.413	0.361	0.454	0.422
149.5	0.413	0.361	0.452	0.425
150	0.41	0.361	0.452	0.42
150.5	0.41	0.359	0.449	0.42

151	0.413	0.359	0.449	0.42
151.5	0.415	0.359	0.452	0.422
152	0.413	0.361	0.452	0.422
152.5	0.41	0.359	0.449	0.42
153	0.41	0.359	0.447	0.42
153.5	0.41	0.356	0.449	0.422
154	0.413	0.359	0.449	0.422
154.5	0.413	0.359	0.449	0.422
155	0.41	0.359	0.449	0.42
155.5	0.41	0.359	0.447	0.42
156	0.413	0.359	0.449	0.42
156.5	0.41	0.359	0.449	0.42
157	0.41	0.361	0.449	0.422
157.5	0.41	0.359	0.452	0.42
158	0.41	0.359	0.449	0.422
158.5	0.41	0.359	0.452	0.422
159	0.41	0.361	0.454	0.422
159.5	0.41	0.361	0.452	0.422
160	0.41	0.359	0.452	0.422
160.5	0.41	0.359	0.452	0.422
161	0.41	0.359	0.452	0.42
161.5	0.413	0.359	0.452	0.422
162	0.41	0.359	0.452	0.422
162.5	0.41	0.356	0.452	0.42
163	0.41	0.356	0.449	0.42
163.5	0.41	0.359	0.452	0.42
164	0.41	0.359	0.452	0.422
164.5	0.41	0.359	0.452	0.42
165	0.41	0.359	0.452	0.42
165.5	0.41	0.359	0.449	0.42
166	0.41	0.359	0.452	0.42
166.5	0.41	0.359	0.452	0.42
167	0.425	0.369	0.469	0.432
167.5	0.439	0.381	0.486	0.447

168	0.454	0.393	0.503	0.461
168.5	0.435	0.376	0.483	0.447
169	0.415	0.359	0.464	0.43
169.5	0.413	0.359	0.461	0.427
170	0.41	0.359	0.459	0.427
170.5	0.413	0.359	0.457	0.427
171	0.413	0.359	0.457	0.427
171.5	0.413	0.359	0.457	0.427
172	0.413	0.359	0.457	0.425
172.5	0.413	0.359	0.454	0.425
173	0.41	0.359	0.454	0.422
173.5	0.413	0.359	0.454	0.422
174	0.41	0.359	0.452	0.422
174.5	0.408	0.359	0.452	0.422
175	0.408	0.356	0.452	0.42
175.5	0.408	0.356	0.452	0.42
176	0.41	0.359	0.452	0.422
176.5	0.41	0.359	0.452	0.422
177	0.408	0.359	0.449	0.42
177.5	0.408	0.359	0.447	0.417
178	0.408	0.356	0.449	0.42
178.5	0.408	0.359	0.449	0.42
179	0.408	0.356	0.449	0.42
179.5	0.405	0.356	0.447	0.417
180	0.405	0.356	0.449	0.417
180.5	0.408	0.356	0.449	0.417
181	0.408	0.359	0.449	0.42
181.5	0.408	0.356	0.449	0.42
182	0.408	0.356	0.449	0.417
182.5	0.408	0.356	0.449	0.42
183	0.408	0.359	0.449	0.42
183.5	0.41	0.359	0.452	0.422
184	0.408	0.359	0.449	0.42
184.5	0.408	0.356	0.447	0.417

185	0.405	0.356	0.447	0.417
185.5	0.408	0.359	0.447	0.417
186	0.408	0.359	0.449	0.42
186.5	0.405	0.359	0.447	0.417
187	0.405	0.356	0.447	0.417
187.5	0.408	0.359	0.447	0.417
188	0.408	0.359	0.447	0.42
188.5	0.408	0.359	0.449	0.42
189	0.408	0.359	0.447	0.42
189.5	0.405	0.356	0.447	0.417
190	0.405	0.356	0.447	0.417
190.5	0.408	0.359	0.447	0.42
191	0.408	0.359	0.447	0.42
191.5	0.405	0.356	0.447	0.417
192	0.405	0.356	0.447	0.417
192.5	0.405	0.356	0.444	0.417
193	0.405	0.356	0.447	0.417
193.5	0.405	0.356	0.447	0.42
194	0.405	0.356	0.447	0.417
194.5	0.405	0.356	0.447	0.417
195	0.405	0.356	0.444	0.417
195.5	0.405	0.356	0.447	0.417
196	0.405	0.356	0.447	0.417
196.5	0.405	0.356	0.444	0.417
197	0.42	0.366	0.461	0.43
197.5	0.435	0.376	0.479	0.442
198	0.449	0.388	0.498	0.457
198.5	0.425	0.376	0.474	0.444
199	0.4	0.364	0.447	0.432
199.5	0.398	0.361	0.447	0.43
200	0.4	0.361	0.447	0.427
200.5	0.4	0.361	0.444	0.425
201	0.4	0.361	0.447	0.425
201.5	0.403	0.359	0.447	0.425

202	0.4	0.359	0.447	0.422
202.5	0.4	0.356	0.444	0.42
203	0.403	0.356	0.444	0.417
203.5	0.4	0.356	0.444	0.42
204	0.405	0.356	0.444	0.42
204.5	0.403	0.356	0.444	0.42
205	0.403	0.356	0.442	0.417
205.5	0.403	0.359	0.444	0.417
206	0.403	0.356	0.444	0.417
206.5	0.403	0.359	0.444	0.417
207	0.403	0.356	0.444	0.417
207.5	0.403	0.356	0.444	0.415
208	0.403	0.356	0.442	0.417
208.5	0.405	0.356	0.444	0.417
209	0.405	0.356	0.444	0.417
209.5	0.403	0.356	0.444	0.417
210	0.403	0.356	0.444	0.415
210.5	0.403	0.356	0.442	0.415
211	0.403	0.356	0.444	0.415
211.5	0.405	0.356	0.444	0.417
212	0.403	0.356	0.442	0.415
212.5	0.403	0.356	0.442	0.415
213	0.403	0.356	0.442	0.415
213.5	0.403	0.356	0.444	0.415
214	0.403	0.356	0.444	0.415
214.5	0.403	0.354	0.442	0.413
215	0.4	0.354	0.442	0.415
215.5	0.403	0.356	0.444	0.415
216	0.403	0.354	0.444	0.415
216.5	0.403	0.356	0.444	0.415
217	0.4	0.356	0.442	0.415
217.5	0.4	0.356	0.442	0.413
218	0.4	0.356	0.442	0.413
218.5	0.403	0.356	0.444	0.413

219	0.403	0.356	0.444	0.415
219.5	0.403	0.354	0.442	0.413
220	0.4	0.354	0.442	0.413
220.5	0.4	0.354	0.444	0.415
221	0.4	0.354	0.442	0.415
221.5	0.4	0.354	0.442	0.415
222	0.4	0.356	0.442	0.413
222.5	0.4	0.354	0.442	0.413
223	0.4	0.354	0.442	0.413
223.5	0.403	0.356	0.444	0.415
224	0.403	0.356	0.444	0.415
224.5	0.403	0.356	0.442	0.413
225	0.403	0.356	0.444	0.413
225.5	0.403	0.356	0.444	0.415
226	0.403	0.356	0.444	0.415
226.5	0.403	0.356	0.442	0.415
227	0.415	0.366	0.457	0.427
227.5	0.427	0.376	0.474	0.439
228	0.442	0.386	0.491	0.452
228.5	0.422	0.366	0.461	0.427
229	0.4	0.344	0.432	0.403
229.5	0.4	0.347	0.432	0.403
230	0.403	0.347	0.435	0.405
230.5	0.403	0.349	0.435	0.408
231	0.403	0.349	0.437	0.405
231.5	0.4	0.349	0.437	0.408
232	0.403	0.352	0.437	0.408
232.5	0.403	0.352	0.439	0.41
233	0.403	0.352	0.439	0.41
233.5	0.403	0.352	0.439	0.41
234	0.4	0.352	0.439	0.41
234.5	0.403	0.352	0.439	0.41
235	0.405	0.354	0.439	0.41
235.5	0.403	0.354	0.442	0.41

236	0.403	0.354	0.439	0.41
236.5	0.4	0.352	0.439	0.41
237	0.403	0.354	0.439	0.41
237.5	0.403	0.354	0.442	0.413
238	0.403	0.356	0.442	0.413
238.5	0.403	0.356	0.442	0.413
239	0.403	0.354	0.442	0.413
239.5	0.403	0.354	0.442	0.413
240	0.403	0.356	0.442	0.413
240.5	0.403	0.356	0.442	0.413
241	0.4	0.356	0.439	0.413
241.5	0.4	0.356	0.439	0.415
242	0.4	0.356	0.442	0.415
242.5	0.403	0.356	0.442	0.413
243	0.403	0.356	0.442	0.415
243.5	0.4	0.354	0.439	0.413
244	0.4	0.354	0.439	0.413
244.5	0.4	0.356	0.439	0.413
245	0.403	0.356	0.439	0.413
245.5	0.403	0.356	0.439	0.413
246	0.4	0.354	0.439	0.413
246.5	0.403	0.354	0.439	0.413
247	0.4	0.354	0.439	0.413
247.5	0.403	0.356	0.442	0.415
248	0.403	0.356	0.442	0.415
248.5	0.4	0.354	0.439	0.413
249	0.4	0.354	0.442	0.413
249.5	0.4	0.354	0.442	0.413
250	0.4	0.354	0.442	0.413
250.5	0.4	0.354	0.442	0.413
251	0.4	0.354	0.442	0.413
251.5	0.4	0.354	0.439	0.413
252	0.4	0.352	0.439	0.413
252.5	0.4	0.354	0.442	0.413

253	0.4	0.354	0.439	0.413
253.5	0.4	0.354	0.439	0.413
254	0.398	0.352	0.437	0.413
254.5	0.4	0.352	0.439	0.413
255	0.4	0.352	0.439	0.415
255.5	0.4	0.354	0.439	0.413
256	0.4	0.354	0.437	0.41
256.5	0.398	0.354	0.437	0.413
257	0.41	0.364	0.452	0.422
257.5	0.422	0.374	0.469	0.435
258	0.437	0.383	0.486	0.447
258.5	0.415	0.374	0.457	0.432
259	0.393	0.361	0.427	0.415
259.5	0.393	0.361	0.43	0.415
260	0.396	0.361	0.432	0.415
260.5	0.396	0.361	0.435	0.415
261	0.396	0.359	0.435	0.413
261.5	0.398	0.359	0.435	0.413
262	0.398	0.359	0.435	0.415
262.5	0.398	0.359	0.437	0.415
263	0.4	0.356	0.437	0.413
263.5	0.398	0.356	0.435	0.413
264	0.398	0.356	0.437	0.413
264.5	0.398	0.354	0.439	0.413
265	0.398	0.356	0.439	0.413
265.5	0.398	0.354	0.437	0.413
266	0.398	0.354	0.437	0.41
266.5	0.396	0.354	0.437	0.41
267	0.398	0.354	0.437	0.41
267.5	0.398	0.354	0.439	0.413
268	0.398	0.354	0.439	0.41
268.5	0.398	0.354	0.439	0.41
269	0.398	0.354	0.437	0.41
269.5	0.398	0.356	0.439	0.413

270	0.4	0.356	0.439	0.413
270.5	0.398	0.356	0.439	0.41
271	0.398	0.354	0.439	0.41
271.5	0.398	0.354	0.437	0.41
272	0.398	0.354	0.437	0.413
272.5	0.398	0.354	0.437	0.413
273	0.398	0.354	0.437	0.413
273.5	0.398	0.354	0.437	0.41
274	0.398	0.354	0.437	0.41
274.5	0.398	0.354	0.437	0.413
275	0.4	0.356	0.437	0.415
275.5	0.4	0.354	0.437	0.413
276	0.4	0.354	0.437	0.413
276.5	0.398	0.354	0.437	0.413
277	0.398	0.356	0.437	0.413
277.5	0.4	0.354	0.439	0.41
278	0.4	0.354	0.439	0.413
278.5	0.4	0.354	0.437	0.41
279	0.4	0.354	0.437	0.408
279.5	0.4	0.354	0.439	0.41
280	0.4	0.354	0.437	0.41
280.5	0.398	0.354	0.437	0.41
281	0.398	0.352	0.437	0.41
281.5	0.398	0.354	0.437	0.41
282	0.398	0.354	0.437	0.41
282.5	0.398	0.354	0.437	0.41
283	0.398	0.354	0.437	0.41
283.5	0.398	0.354	0.437	0.41
284	0.398	0.352	0.437	0.41
284.5	0.398	0.354	0.437	0.41
285	0.398	0.354	0.437	0.41
285.5	0.396	0.352	0.435	0.41
286	0.396	0.354	0.435	0.41
286.5	0.396	0.352	0.437	0.41

287	0.408	0.359	0.452	0.42
287.5	0.42	0.369	0.466	0.43
288	0.435	0.378	0.481	0.442
288.5	0.413	0.364	0.464	0.435
289	0.388	0.349	0.444	0.427
289.5	0.388	0.352	0.444	0.427
290	0.388	0.352	0.442	0.425
290.5	0.391	0.352	0.439	0.422
291	0.391	0.352	0.439	0.42
291.5	0.393	0.352	0.439	0.42
292	0.393	0.354	0.439	0.417
292.5	0.393	0.354	0.437	0.417
293	0.396	0.354	0.439	0.415
293.5	0.393	0.352	0.437	0.415
294	0.396	0.352	0.437	0.415
294.5	0.396	0.354	0.437	0.415
295	0.396	0.354	0.437	0.415
295.5	0.396	0.352	0.437	0.413
296	0.396	0.352	0.437	0.413
296.5	0.398	0.352	0.437	0.413
297	0.398	0.354	0.437	0.413
297.5	0.396	0.352	0.435	0.413
298	0.396	0.352	0.437	0.413
298.5	0.396	0.352	0.437	0.413
299	0.396	0.352	0.437	0.413
299.5	0.398	0.354	0.437	0.413
300	0.396	0.354	0.437	0.41
300.5	0.396	0.352	0.437	0.41
301	0.396	0.352	0.437	0.41
301.5	0.398	0.354	0.437	0.41
302	0.398	0.354	0.439	0.41
302.5	0.396	0.352	0.435	0.41
303	0.396	0.352	0.437	0.41
303.5	0.396	0.352	0.435	0.41

304	0.398	0.352	0.437	0.41
304.5	0.398	0.352	0.437	0.41
305	0.396	0.352	0.437	0.41
305.5	0.396	0.352	0.435	0.41
306	0.396	0.352	0.435	0.41
306.5	0.398	0.354	0.437	0.41
307	0.398	0.354	0.437	0.41
307.5	0.396	0.354	0.435	0.41
308	0.398	0.354	0.435	0.41
308.5	0.398	0.354	0.437	0.41
309	0.398	0.354	0.437	0.41
309.5	0.398	0.354	0.435	0.41
310	0.398	0.354	0.435	0.408
310.5	0.398	0.354	0.435	0.408
311	0.398	0.354	0.435	0.41
311.5	0.4	0.354	0.437	0.413
312	0.398	0.354	0.437	0.41
312.5	0.398	0.352	0.437	0.408
313	0.398	0.352	0.437	0.408
313.5	0.398	0.352	0.437	0.408
314	0.398	0.354	0.437	0.41
314.5	0.398	0.354	0.437	0.408
315	0.398	0.352	0.437	0.408
315.5	0.396	0.352	0.435	0.41
316	0.398	0.352	0.435	0.41
316.5	0.408	0.359	0.447	0.42
317	0.42	0.369	0.461	0.43
317.5	0.432	0.378	0.476	0.439
318	0.405	0.364	0.449	0.417
318.5	0.378	0.349	0.422	0.393
319	0.381	0.349	0.422	0.393
319.5	0.383	0.352	0.425	0.393
320	0.386	0.352	0.427	0.396
320.5	0.386	0.352	0.425	0.398

321	0.388	0.352	0.427	0.398
321.5	0.388	0.352	0.43	0.4
322	0.391	0.352	0.43	0.4
322.5	0.391	0.354	0.432	0.403
323	0.391	0.352	0.43	0.403
323.5	0.391	0.352	0.432	0.405
324	0.393	0.352	0.432	0.405
324.5	0.393	0.354	0.432	0.405
325	0.393	0.354	0.432	0.405
325.5	0.396	0.354	0.435	0.405
326	0.393	0.354	0.432	0.405
326.5	0.396	0.352	0.432	0.408
327	0.396	0.352	0.435	0.408
327.5	0.396	0.352	0.435	0.405
328	0.393	0.352	0.432	0.408
328.5	0.393	0.352	0.435	0.408
329	0.393	0.352	0.435	0.408
329.5	0.396	0.354	0.435	0.408
330	0.396	0.352	0.435	0.408
330.5	0.393	0.352	0.435	0.408
331	0.396	0.354	0.432	0.408
331.5	0.393	0.352	0.435	0.408
332	0.396	0.354	0.435	0.408
332.5	0.396	0.354	0.435	0.408
333	0.396	0.354	0.435	0.408
333.5	0.396	0.354	0.435	0.408
334	0.396	0.352	0.435	0.41
334.5	0.396	0.354	0.435	0.41
335	0.396	0.352	0.435	0.408
335.5	0.393	0.352	0.435	0.41
336	0.396	0.352	0.435	0.408
336.5	0.396	0.352	0.435	0.41
337	0.396	0.354	0.435	0.41
337.5	0.396	0.352	0.435	0.41

338	0.393	0.349	0.432	0.408
338.5	0.393	0.352	0.432	0.408
339	0.396	0.352	0.432	0.408
339.5	0.396	0.352	0.432	0.408
340	0.396	0.352	0.432	0.408
340.5	0.393	0.352	0.432	0.408
341	0.393	0.352	0.432	0.405
341.5	0.393	0.349	0.432	0.408
342	0.396	0.352	0.435	0.408
342.5	0.396	0.352	0.432	0.408
343	0.393	0.352	0.43	0.408
343.5	0.393	0.352	0.432	0.408
344	0.393	0.352	0.432	0.408
344.5	0.396	0.352	0.432	0.408
345	0.393	0.352	0.432	0.408
345.5	0.396	0.352	0.435	0.408
346	0.393	0.352	0.432	0.408
346.5	0.403	0.359	0.444	0.417
347	0.415	0.366	0.457	0.427
347.5	0.427	0.376	0.471	0.437
348	0.415	0.374	0.454	0.417
348.5	0.403	0.371	0.435	0.398
349	0.403	0.371	0.435	0.398
349.5	0.4	0.369	0.432	0.398
350	0.398	0.366	0.432	0.4
350.5	0.398	0.364	0.432	0.4
351	0.398	0.364	0.432	0.403
351.5	0.398	0.361	0.432	0.403
352	0.398	0.359	0.435	0.403
352.5	0.396	0.359	0.435	0.403
353	0.398	0.359	0.432	0.403
353.5	0.398	0.356	0.432	0.403
354	0.396	0.356	0.432	0.403
354.5	0.396	0.354	0.432	0.403

355	0.393	0.354	0.432	0.403
355.5	0.396	0.354	0.435	0.405
356	0.396	0.354	0.435	0.405
356.5	0.396	0.354	0.432	0.405
357	0.396	0.354	0.432	0.405
357.5	0.396	0.354	0.432	0.405
358	0.396	0.354	0.432	0.405
358.5	0.396	0.354	0.432	0.405
359	0.393	0.354	0.432	0.405
359.5	0.393	0.352	0.432	0.405
360	0.393	0.352	0.432	0.405
360.5	0.393	0.352	0.432	0.408
361	0.393	0.352	0.432	0.405
361.5	0.393	0.352	0.432	0.405
362	0.393	0.352	0.432	0.408
362.5	0.393	0.352	0.432	0.405
363	0.393	0.352	0.432	0.405
363.5	0.393	0.352	0.432	0.405
364	0.393	0.352	0.432	0.405
364.5	0.393	0.352	0.432	0.405
365	0.393	0.352	0.43	0.408
365.5	0.393	0.352	0.43	0.408
366	0.393	0.352	0.43	0.405
366.5	0.393	0.352	0.43	0.405
367	0.393	0.352	0.43	0.405
367.5	0.393	0.352	0.43	0.405
368	0.393	0.352	0.432	0.405
368.5	0.393	0.352	0.432	0.408
369	0.393	0.352	0.43	0.405
369.5	0.393	0.352	0.43	0.405
370	0.393	0.352	0.43	0.408
370.5	0.393	0.352	0.432	0.408
371	0.393	0.352	0.432	0.408
371.5	0.393	0.352	0.432	0.408

372	0.393	0.349	0.432	0.408
372.5	0.393	0.352	0.432	0.408
373	0.393	0.354	0.435	0.408
373.5	0.393	0.352	0.432	0.408
374	0.393	0.352	0.432	0.405
374.5	0.393	0.352	0.432	0.405
375	0.393	0.352	0.432	0.405
375.5	0.396	0.352	0.432	0.408
376	0.393	0.352	0.432	0.408
376.5	0.403	0.359	0.442	0.415
377	0.413	0.366	0.454	0.422
377.5	0.425	0.374	0.466	0.432
378	0.41	0.361	0.444	0.417
378.5	0.393	0.347	0.42	0.4
379	0.393	0.347	0.42	0.4
379.5	0.391	0.347	0.422	0.403
380	0.391	0.349	0.422	0.403
380.5	0.393	0.349	0.425	0.405
381	0.393	0.349	0.425	0.405
381.5	0.393	0.349	0.425	0.405
382	0.393	0.349	0.427	0.405
382.5	0.393	0.349	0.427	0.403
383	0.393	0.349	0.427	0.405
383.5	0.393	0.349	0.427	0.405
384	0.393	0.349	0.427	0.408
384.5	0.393	0.349	0.427	0.405
385	0.393	0.349	0.43	0.405
385.5	0.391	0.349	0.43	0.405
386	0.393	0.349	0.43	0.405
386.5	0.393	0.349	0.43	0.405
387	0.391	0.349	0.43	0.405
387.5	0.391	0.349	0.43	0.405
388	0.391	0.349	0.43	0.408
388.5	0.393	0.352	0.43	0.408

389	0.393	0.349	0.43	0.408
389.5	0.393	0.349	0.427	0.405
390	0.391	0.349	0.427	0.405
390.5	0.393	0.349	0.43	0.408
391	0.393	0.352	0.43	0.405
391.5	0.393	0.352	0.427	0.405
392	0.391	0.349	0.427	0.405
392.5	0.391	0.352	0.43	0.405
393	0.393	0.352	0.43	0.405
393.5	0.393	0.352	0.432	0.405
394	0.391	0.352	0.43	0.405
394.5	0.391	0.352	0.43	0.403
395	0.393	0.352	0.43	0.405
395.5	0.393	0.352	0.43	0.405
396	0.393	0.352	0.43	0.405
396.5	0.393	0.349	0.43	0.405
397	0.393	0.349	0.43	0.403
397.5	0.391	0.349	0.43	0.405
398	0.393	0.352	0.432	0.405
398.5	0.391	0.349	0.43	0.405
399	0.391	0.349	0.43	0.405
399.5	0.393	0.349	0.43	0.403
400	0.391	0.349	0.43	0.405
400.5	0.393	0.349	0.43	0.405
401	0.391	0.352	0.43	0.405
401.5	0.391	0.352	0.43	0.405
402	0.393	0.352	0.43	0.408
402.5	0.393	0.349	0.43	0.405
403	0.393	0.352	0.432	0.405
403.5	0.393	0.349	0.43	0.408
404	0.391	0.352	0.43	0.405
404.5	0.391	0.349	0.43	0.405
405	0.391	0.352	0.432	0.408
405.5	0.393	0.352	0.432	0.408

406	0.391	0.352	0.43	0.408
406.5	0.393	0.352	0.43	0.405
407	0.4	0.356	0.439	0.413
407.5	0.41	0.364	0.449	0.42
408	0.42	0.371	0.461	0.43
408.5	0.415	0.366	0.454	0.42
409	0.408	0.361	0.444	0.408
409.5	0.405	0.361	0.442	0.408
410	0.403	0.359	0.439	0.408
410.5	0.4	0.356	0.437	0.405
411	0.4	0.356	0.437	0.405
411.5	0.4	0.356	0.435	0.408
412	0.398	0.354	0.437	0.408
412.5	0.396	0.354	0.435	0.405
413	0.396	0.352	0.432	0.405
413.5	0.396	0.352	0.435	0.408
414	0.396	0.354	0.435	0.408
414.5	0.396	0.354	0.435	0.408
415	0.396	0.352	0.432	0.405
415.5	0.393	0.352	0.43	0.405
416	0.393	0.352	0.432	0.405
416.5	0.396	0.354	0.432	0.408
417	0.393	0.352	0.432	0.408
417.5	0.393	0.352	0.43	0.405
418	0.396	0.352	0.43	0.405
418.5	0.396	0.354	0.43	0.405
419	0.393	0.354	0.43	0.405
419.5	0.393	0.352	0.432	0.405
420	0.393	0.352	0.43	0.403
420.5	0.393	0.352	0.43	0.403
421	0.393	0.352	0.43	0.405
421.5	0.391	0.352	0.43	0.405
422	0.391	0.352	0.43	0.405
422.5	0.391	0.352	0.427	0.405

423	0.391	0.349	0.43	0.403
423.5	0.393	0.349	0.43	0.405
424	0.393	0.352	0.43	0.408
424.5	0.391	0.349	0.43	0.405
425	0.391	0.349	0.43	0.405
425.5	0.391	0.349	0.43	0.405
426	0.391	0.349	0.43	0.405
426.5	0.391	0.352	0.43	0.405
427	0.391	0.349	0.43	0.405
427.5	0.391	0.349	0.43	0.405
428	0.391	0.349	0.43	0.405
428.5	0.391	0.349	0.43	0.405
429	0.391	0.352	0.43	0.405
429.5	0.391	0.352	0.43	0.405
430	0.388	0.349	0.427	0.403
430.5	0.388	0.349	0.427	0.405
431	0.388	0.349	0.427	0.405
431.5	0.391	0.349	0.427	0.405
432	0.388	0.349	0.427	0.405
432.5	0.388	0.349	0.427	0.403
433	0.388	0.349	0.427	0.403
433.5	0.388	0.349	0.427	0.403
434	0.391	0.349	0.427	0.405
434.5	0.391	0.349	0.43	0.403
435	0.388	0.349	0.427	0.403
435.5	0.388	0.349	0.427	0.403
436	0.391	0.349	0.427	0.403
436.5	0.391	0.349	0.43	0.405
437	0.398	0.354	0.439	0.413
437.5	0.408	0.361	0.449	0.42
438	0.417	0.369	0.459	0.427
438.5	0.405	0.369	0.437	0.42
439	0.391	0.366	0.415	0.413
439.5	0.393	0.364	0.417	0.41

440	0.393	0.364	0.417	0.41
440.5	0.393	0.364	0.42	0.41
441	0.393	0.364	0.422	0.413
441.5	0.393	0.361	0.422	0.413
442	0.393	0.361	0.422	0.413
442.5	0.393	0.361	0.425	0.413
443	0.398	0.364	0.427	0.415
443.5	0.396	0.364	0.427	0.413
444	0.396	0.361	0.427	0.413
444.5	0.398	0.361	0.427	0.413
445	0.398	0.361	0.427	0.413
445.5	0.398	0.361	0.43	0.413
446	0.398	0.361	0.427	0.413
446.5	0.398	0.364	0.427	0.413
447	0.396	0.361	0.427	0.41
447.5	0.398	0.361	0.427	0.413
448	0.398	0.361	0.43	0.413
448.5	0.398	0.364	0.43	0.41
449	0.398	0.361	0.43	0.41
449.5	0.398	0.361	0.43	0.41
450	0.398	0.361	0.43	0.413
450.5	0.398	0.361	0.432	0.413
451	0.396	0.361	0.432	0.413
451.5	0.396	0.361	0.432	0.41
452	0.396	0.361	0.43	0.41
452.5	0.396	0.359	0.43	0.41
453	0.396	0.359	0.43	0.413
453.5	0.396	0.361	0.43	0.41
454	0.396	0.361	0.432	0.41
454.5	0.396	0.361	0.432	0.413
455	0.396	0.361	0.432	0.413
455.5	0.396	0.361	0.432	0.413
456	0.396	0.361	0.43	0.413
456.5	0.396	0.361	0.43	0.41

457	0.396	0.361	0.43	0.413
457.5	0.396	0.361	0.43	0.413
458	0.396	0.361	0.432	0.413
458.5	0.398	0.359	0.43	0.413
459	0.396	0.361	0.432	0.413
459.5	0.396	0.361	0.432	0.413
460	0.398	0.361	0.432	0.413
460.5	0.398	0.361	0.432	0.413
461	0.396	0.361	0.432	0.413
461.5	0.396	0.361	0.43	0.413
462	0.398	0.361	0.432	0.413
462.5	0.398	0.364	0.432	0.413
463	0.398	0.364	0.432	0.415
463.5	0.396	0.361	0.43	0.413
464	0.396	0.364	0.43	0.413
464.5	0.396	0.361	0.43	0.415
465	0.398	0.364	0.432	0.415
465.5	0.398	0.364	0.432	0.415
466	0.398	0.361	0.432	0.415
466.5	0.398	0.361	0.432	0.413
467	0.403	0.364	0.439	0.417
467.5	0.41	0.366	0.447	0.422
468	0.417	0.371	0.457	0.427
468.5	0.408	0.369	0.442	0.42
469	0.396	0.364	0.425	0.413
469.5	0.396	0.364	0.425	0.413
470	0.396	0.364	0.427	0.413
470.5	0.396	0.364	0.427	0.415
471	0.396	0.361	0.427	0.413
471.5	0.396	0.364	0.427	0.413
472	0.396	0.361	0.43	0.413
472.5	0.396	0.364	0.43	0.413
473	0.396	0.361	0.43	0.413
473.5	0.396	0.361	0.43	0.41

474	0.396	0.361	0.43	0.41
474.5	0.396	0.361	0.43	0.413
475	0.396	0.361	0.432	0.413
475.5	0.396	0.361	0.43	0.413
476	0.393	0.359	0.43	0.41
476.5	0.396	0.361	0.43	0.413
477	0.396	0.361	0.43	0.413
477.5	0.396	0.364	0.43	0.415
478	0.396	0.361	0.43	0.413
478.5	0.396	0.361	0.43	0.415
479	0.396	0.359	0.43	0.413
479.5	0.396	0.361	0.43	0.415
480	0.393	0.359	0.43	0.413
480.5	0.393	0.359	0.43	0.41
481	0.393	0.359	0.43	0.413
481.5	0.393	0.359	0.43	0.413
482	0.396	0.359	0.43	0.413
482.5	0.396	0.361	0.432	0.413
483	0.393	0.359	0.43	0.413
483.5	0.396	0.359	0.43	0.413
484	0.396	0.359	0.43	0.41
484.5	0.396	0.361	0.432	0.413
485	0.396	0.361	0.43	0.413
485.5	0.396	0.361	0.43	0.41
486	0.396	0.361	0.43	0.413
486.5	0.396	0.361	0.43	0.413
487	0.396	0.361	0.43	0.413
487.5	0.396	0.361	0.43	0.413
488	0.396	0.361	0.43	0.41
488.5	0.396	0.361	0.43	0.41
489	0.396	0.361	0.43	0.41
489.5	0.396	0.361	0.43	0.413
490	0.396	0.361	0.43	0.413
490.5	0.393	0.361	0.43	0.41

491	0.393	0.359	0.43	0.413
491.5	0.396	0.359	0.427	0.413
492	0.396	0.361	0.43	0.413
492.5	0.396	0.361	0.43	0.413
493	0.396	0.361	0.43	0.413
493.5	0.393	0.361	0.43	0.41
494	0.393	0.361	0.43	0.41
494.5	0.393	0.361	0.432	0.41
495	0.393	0.361	0.432	0.41
495.5	0.393	0.361	0.432	0.41
496	0.393	0.361	0.43	0.41
496.5	0.393	0.359	0.427	0.41
497	0.393	0.361	0.427	0.41
497.5	0.391	0.361	0.427	0.408
498	0.391	0.359	0.427	0.408
498.5	0.391	0.359	0.427	0.408
499	0.391	0.359	0.427	0.408
499.5	0.391	0.359	0.425	0.408
500	0.391	0.359	0.425	0.408
500.5	0.388	0.359	0.425	0.405
501	0.388	0.359	0.425	0.405
501.5	0.388	0.356	0.422	0.405
502	0.388	0.356	0.422	0.405
502.5	0.388	0.356	0.422	0.405
503	0.388	0.356	0.422	0.405
503.5	0.386	0.356	0.42	0.403
504	0.386	0.356	0.42	0.403
504.5	0.386	0.356	0.42	0.403
505	0.403	0.366	0.437	0.417
505.5	0.422	0.376	0.454	0.432
506	0.442	0.386	0.471	0.447
506.5	0.403	0.361	0.435	0.413
507	0.364	0.334	0.398	0.376
507.5	0.359	0.317	0.393	0.381

508	0.361	0.317	0.393	0.381
508.5	0.361	0.317	0.393	0.381
509	0.359	0.317	0.393	0.381
509.5	0.359	0.317	0.393	0.381
510	0.359	0.32	0.396	0.381
510.5	0.359	0.32	0.396	0.381
511	0.361	0.32	0.393	0.381
511.5	0.361	0.32	0.393	0.381
512	0.361	0.317	0.393	0.381
512.5	0.361	0.317	0.393	0.381
513	0.361	0.32	0.393	0.381
513.5	0.361	0.32	0.393	0.381
514	0.359	0.32	0.393	0.381
514.5	0.359	0.32	0.393	0.381
515	0.361	0.32	0.393	0.381
515.5	0.361	0.32	0.393	0.381
516	0.361	0.32	0.393	0.381
516.5	0.361	0.32	0.393	0.381
517	0.391	0.342	0.422	0.405
517.5	0.42	0.364	0.452	0.43
518	0.452	0.388	0.483	0.457
518.5	0.41	0.359	0.442	0.422
519	0.369	0.327	0.4	0.388
519.5	0.374	0.342	0.396	0.378
520	0.374	0.342	0.396	0.378
520.5	0.371	0.342	0.393	0.378
521	0.371	0.342	0.393	0.378
521.5	0.371	0.342	0.396	0.376
522	0.371	0.342	0.396	0.376
522.5	0.371	0.342	0.393	0.378
523	0.371	0.342	0.393	0.378
523.5	0.371	0.342	0.393	0.378
524	0.371	0.342	0.393	0.378
524.5	0.371	0.342	0.393	0.378

525	0.371	0.342	0.393	0.378
525.5	0.371	0.339	0.396	0.378
526	0.371	0.339	0.396	0.378
526.5	0.371	0.339	0.393	0.378
527	0.371	0.339	0.393	0.378
527.5	0.371	0.339	0.393	0.378
528	0.371	0.339	0.393	0.378
528.5	0.371	0.339	0.393	0.376
529	0.371	0.339	0.393	0.376
529.5	0.371	0.339	0.393	0.376
530	0.371	0.339	0.393	0.376
530.5	0.371	0.339	0.393	0.378
531	0.398	0.354	0.427	0.405
531.5	0.427	0.369	0.461	0.435
532	0.457	0.383	0.496	0.464
532.5	0.42	0.356	0.447	0.427
533	0.381	0.33	0.398	0.388
533.5	0.381	0.33	0.398	0.388
534	0.381	0.327	0.398	0.388
534.5	0.381	0.327	0.398	0.388
535	0.381	0.33	0.398	0.388
535.5	0.381	0.327	0.398	0.388
536	0.381	0.327	0.398	0.388
536.5	0.381	0.327	0.398	0.388
537	0.381	0.327	0.398	0.388
537.5	0.381	0.327	0.398	0.388
538	0.378	0.327	0.398	0.388
538.5	0.378	0.327	0.398	0.388
539	0.378	0.327	0.398	0.388
539.5	0.378	0.327	0.398	0.388
540	0.378	0.327	0.398	0.386
540.5	0.378	0.327	0.398	0.386
541	0.378	0.327	0.398	0.388
541.5	0.378	0.327	0.398	0.388

542	0.378	0.327	0.398	0.388
542.5	0.378	0.327	0.398	0.388
543	0.378	0.327	0.398	0.386
543.5	0.378	0.327	0.398	0.386
544	0.376	0.327	0.398	0.386
544.5	0.376	0.327	0.398	0.386
545	0.376	0.327	0.398	0.386
545.5	0.376	0.327	0.398	0.386
546	0.376	0.327	0.398	0.386
546.5	0.376	0.327	0.398	0.386
547	0.376	0.327	0.396	0.386
547.5	0.376	0.327	0.396	0.386
548	0.403	0.347	0.43	0.413
548.5	0.432	0.366	0.464	0.439
549	0.461	0.386	0.5	0.466
549.5	0.422	0.366	0.449	0.427
550	0.383	0.347	0.396	0.388
550.5	0.374	0.344	0.383	0.383
551	0.364	0.332	0.374	0.371
551.5	0.374	0.342	0.386	0.383
552	0.374	0.342	0.386	0.383
552.5	0.374	0.342	0.386	0.383
553	0.374	0.342	0.386	0.383
553.5	0.374	0.342	0.386	0.383
554	0.371	0.339	0.386	0.383
554.5	0.371	0.339	0.386	0.383
555	0.374	0.339	0.386	0.381
555.5	0.374	0.339	0.386	0.381
556	0.374	0.342	0.386	0.381
556.5	0.374	0.342	0.386	0.381
557	0.374	0.339	0.386	0.381
557.5	0.374	0.339	0.386	0.381
558	0.374	0.339	0.386	0.381
558.5	0.374	0.339	0.386	0.381

559	0.371	0.339	0.386	0.381
559.5	0.371	0.339	0.386	0.381
560	0.371	0.339	0.388	0.381
560.5	0.371	0.339	0.388	0.381
561	0.371	0.337	0.388	0.381
561.5	0.371	0.337	0.388	0.381
562	0.371	0.337	0.388	0.381
562.5	0.371	0.337	0.388	0.381
563	0.371	0.339	0.388	0.381
563.5	0.371	0.339	0.388	0.381
564	0.371	0.339	0.388	0.381
564.5	0.371	0.339	0.388	0.381
565	0.371	0.337	0.388	0.381
565.5	0.371	0.337	0.388	0.381
566	0.371	0.337	0.388	0.381
566.5	0.371	0.337	0.388	0.381
567	0.371	0.337	0.388	0.381
567.5	0.371	0.337	0.388	0.381
568	0.4	0.354	0.425	0.408
568.5	0.43	0.371	0.464	0.437
569	0.461	0.388	0.503	0.466
569.5	0.417	0.364	0.447	0.425
570	0.371	0.337	0.388	0.381
570.5	0.376	0.334	0.393	0.391
571	0.376	0.334	0.393	0.391
571.5	0.376	0.334	0.393	0.388
572	0.376	0.334	0.393	0.388
572.5	0.376	0.334	0.393	0.388
573	0.376	0.334	0.393	0.388
573.5	0.376	0.334	0.393	0.388
574	0.376	0.334	0.391	0.388
574.5	0.376	0.334	0.391	0.388
575	0.376	0.334	0.393	0.388
575.5	0.376	0.334	0.393	0.388

576	0.376	0.334	0.393	0.388
576.5	0.374	0.332	0.393	0.388
577	0.374	0.332	0.393	0.388
577.5	0.374	0.332	0.393	0.388
578	0.374	0.332	0.393	0.388
578.5	0.374	0.332	0.393	0.388
579	0.374	0.332	0.393	0.388
579.5	0.376	0.334	0.393	0.386
580	0.376	0.334	0.393	0.386
580.5	0.374	0.332	0.393	0.386
581	0.374	0.332	0.393	0.386
581.5	0.376	0.332	0.393	0.386
582	0.376	0.332	0.393	0.386
582.5	0.374	0.332	0.393	0.386
583	0.374	0.332	0.393	0.386
583.5	0.374	0.332	0.393	0.386
584	0.374	0.332	0.393	0.386
584.5	0.374	0.332	0.393	0.386
585	0.374	0.332	0.393	0.386
585.5	0.374	0.332	0.393	0.383
586	0.374	0.332	0.393	0.383
586.5	0.374	0.334	0.396	0.386
587	0.374	0.334	0.396	0.386
587.5	0.374	0.332	0.396	0.386
588	0.374	0.332	0.396	0.386
588.5	0.374	0.332	0.396	0.386
589	0.374	0.332	0.396	0.386
589.5	0.374	0.332	0.393	0.386
590	0.374	0.332	0.393	0.386
590.5	0.374	0.332	0.393	0.386
591	0.374	0.332	0.393	0.386
591.5	0.374	0.332	0.393	0.383
592	0.405	0.349	0.43	0.413
592.5	0.437	0.369	0.469	0.442

593	0.469	0.388	0.508	0.471
593.5	0.422	0.361	0.452	0.43
594	0.374	0.332	0.396	0.386
594.5	0.376	0.342	0.405	0.378
595	0.376	0.342	0.405	0.378
595.5	0.376	0.342	0.405	0.378
596	0.376	0.342	0.405	0.378
596.5	0.376	0.342	0.403	0.378
597	0.376	0.342	0.403	0.378
597.5	0.376	0.342	0.405	0.378
598	0.376	0.342	0.405	0.378
598.5	0.376	0.342	0.403	0.378
599	0.376	0.342	0.403	0.378
599.5	0.376	0.342	0.403	0.378
600	0.376	0.342	0.403	0.378
600.5	0.376	0.342	0.403	0.378
601	0.376	0.339	0.403	0.378
601.5	0.376	0.339	0.403	0.378
602	0.376	0.339	0.403	0.378
602.5	0.376	0.339	0.403	0.378
603	0.376	0.339	0.403	0.378
603.5	0.376	0.339	0.403	0.381
604	0.376	0.339	0.403	0.378
604.5	0.376	0.339	0.403	0.381
605	0.376	0.339	0.403	0.381
605.5	0.374	0.339	0.403	0.381
606	0.374	0.339	0.403	0.381
606.5	0.374	0.337	0.403	0.381
607	0.374	0.337	0.403	0.381
607.5	0.376	0.339	0.403	0.381
608	0.376	0.339	0.403	0.381
608.5	0.374	0.339	0.403	0.381
609	0.374	0.339	0.403	0.381
609.5	0.374	0.339	0.403	0.381

610	0.374	0.339	0.403	0.381
610.5	0.374	0.339	0.403	0.381
611	0.374	0.339	0.403	0.381
611.5	0.374	0.337	0.4	0.381
612	0.374	0.337	0.4	0.381
612.5	0.374	0.337	0.4	0.381
613	0.374	0.337	0.4	0.381
613.5	0.374	0.337	0.4	0.381
614	0.374	0.337	0.4	0.381
614.5	0.374	0.337	0.4	0.381
615	0.374	0.337	0.4	0.381
615.5	0.374	0.337	0.4	0.381
616	0.374	0.337	0.4	0.381
616.5	0.374	0.337	0.4	0.383
617	0.374	0.337	0.4	0.383
617.5	0.374	0.337	0.4	0.381
618	0.374	0.337	0.4	0.381
618.5	0.374	0.337	0.4	0.381
619	0.374	0.337	0.4	0.381
619.5	0.374	0.337	0.4	0.381
620	0.374	0.337	0.4	0.381
620.5	0.374	0.337	0.4	0.381
621	0.405	0.354	0.437	0.41
621.5	0.437	0.371	0.474	0.442
622	0.471	0.388	0.513	0.474
622.5	0.422	0.364	0.457	0.427
623	0.374	0.337	0.4	0.381
623.5	0.376	0.339	0.408	0.388
624	0.376	0.339	0.408	0.388
624.5	0.376	0.339	0.405	0.388
625	0.376	0.339	0.405	0.388
625.5	0.376	0.339	0.405	0.388
626	0.376	0.339	0.405	0.388
626.5	0.376	0.339	0.405	0.388

627	0.376	0.339	0.405	0.388
627.5	0.376	0.339	0.405	0.388
628	0.376	0.339	0.405	0.388
628.5	0.376	0.339	0.405	0.388
629	0.376	0.339	0.405	0.386
629.5	0.376	0.339	0.405	0.386
630	0.376	0.339	0.405	0.388
630.5	0.376	0.339	0.405	0.388
631	0.376	0.339	0.405	0.388
631.5	0.376	0.339	0.405	0.388
632	0.376	0.339	0.405	0.388
632.5	0.376	0.339	0.405	0.388
633	0.376	0.337	0.405	0.388
633.5	0.376	0.337	0.405	0.388
634	0.376	0.337	0.405	0.388
634.5	0.374	0.337	0.403	0.388
635	0.374	0.337	0.403	0.388
635.5	0.374	0.337	0.403	0.388
636	0.374	0.337	0.403	0.388
636.5	0.374	0.337	0.403	0.388
637	0.374	0.337	0.403	0.388
637.5	0.374	0.337	0.403	0.388
638	0.374	0.337	0.403	0.388
638.5	0.374	0.337	0.403	0.388
639	0.374	0.337	0.403	0.388
639.5	0.374	0.337	0.405	0.386
640	0.374	0.337	0.405	0.386
640.5	0.374	0.337	0.405	0.386
641	0.374	0.337	0.405	0.386
641.5	0.374	0.337	0.405	0.388
642	0.374	0.337	0.405	0.388
642.5	0.374	0.337	0.403	0.388
643	0.374	0.337	0.403	0.388
643.5	0.374	0.334	0.405	0.388

644	0.374	0.334	0.405	0.388
644.5	0.374	0.334	0.405	0.388
645	0.374	0.334	0.405	0.388
645.5	0.374	0.334	0.403	0.386
646	0.374	0.334	0.403	0.386
646.5	0.374	0.334	0.403	0.386
647	0.374	0.334	0.403	0.386
647.5	0.374	0.334	0.403	0.386
648	0.374	0.334	0.403	0.386
648.5	0.374	0.334	0.403	0.386
649	0.374	0.334	0.403	0.386
649.5	0.374	0.334	0.403	0.386
650	0.374	0.334	0.403	0.386
650.5	0.374	0.334	0.403	0.386
651	0.374	0.334	0.403	0.386
651.5	0.374	0.334	0.403	0.386
652	0.374	0.334	0.4	0.386
652.5	0.374	0.334	0.4	0.386
653	0.374	0.334	0.403	0.386
653.5	0.374	0.334	0.403	0.386
654	0.374	0.334	0.403	0.386
654.5	0.374	0.334	0.403	0.386
655	0.374	0.334	0.403	0.386
655.5	0.374	0.334	0.403	0.386
656	0.405	0.352	0.439	0.415
656.5	0.439	0.371	0.476	0.447
657	0.474	0.391	0.515	0.479
657.5	0.425	0.364	0.459	0.432
658	0.374	0.334	0.403	0.386
658.5	0.391	0.347	0.403	0.396
659	0.391	0.347	0.403	0.396
659.5	0.391	0.347	0.403	0.396
660	0.391	0.347	0.403	0.396
660.5	0.391	0.347	0.403	0.393

661	0.391	0.347	0.403	0.393
661.5	0.391	0.344	0.403	0.393
662	0.391	0.344	0.403	0.393
662.5	0.391	0.344	0.403	0.393
663	0.391	0.344	0.403	0.393
663.5	0.388	0.344	0.403	0.393
664	0.388	0.344	0.403	0.393
664.5	0.388	0.344	0.4	0.393
665	0.388	0.344	0.403	0.393
665.5	0.388	0.344	0.403	0.393
666	0.388	0.344	0.403	0.393
666.5	0.388	0.344	0.403	0.393
667	0.388	0.344	0.4	0.393
667.5	0.388	0.344	0.4	0.393
668	0.386	0.342	0.4	0.393
668.5	0.386	0.342	0.4	0.393
669	0.386	0.342	0.4	0.393
669.5	0.386	0.342	0.4	0.393
670	0.386	0.342	0.403	0.393
670.5	0.386	0.342	0.403	0.393
671	0.386	0.342	0.4	0.393
671.5	0.386	0.342	0.4	0.393
672	0.386	0.342	0.403	0.393
672.5	0.386	0.342	0.403	0.393
673	0.386	0.342	0.4	0.393
673.5	0.386	0.342	0.4	0.393
674	0.383	0.342	0.4	0.391
674.5	0.383	0.342	0.4	0.391
675	0.386	0.339	0.4	0.391
675.5	0.386	0.339	0.4	0.391
676	0.383	0.339	0.4	0.391
676.5	0.383	0.339	0.4	0.391
677	0.383	0.339	0.4	0.391
677.5	0.383	0.339	0.4	0.391

678	0.383	0.339	0.4	0.391
678.5	0.383	0.339	0.4	0.391
679	0.383	0.339	0.4	0.391
679.5	0.383	0.339	0.4	0.391
680	0.383	0.337	0.4	0.391
680.5	0.383	0.337	0.4	0.391
681	0.381	0.337	0.4	0.391
681.5	0.381	0.337	0.4	0.391
682	0.381	0.337	0.4	0.391
682.5	0.381	0.337	0.4	0.391
683	0.381	0.337	0.4	0.391
683.5	0.381	0.337	0.4	0.391
684	0.381	0.339	0.4	0.391
684.5	0.381	0.337	0.4	0.391
685	0.381	0.337	0.4	0.391
685.5	0.381	0.337	0.4	0.391
686	0.381	0.337	0.4	0.391
686.5	0.381	0.337	0.4	0.391
687	0.381	0.337	0.4	0.391
687.5	0.381	0.337	0.4	0.388
688	0.381	0.337	0.4	0.388
688.5	0.381	0.337	0.4	0.391
689	0.381	0.337	0.4	0.391
689.5	0.381	0.337	0.4	0.391
690	0.381	0.337	0.4	0.391
690.5	0.381	0.337	0.4	0.391
691	0.381	0.337	0.4	0.391
691.5	0.381	0.334	0.4	0.391
692	0.381	0.334	0.4	0.391
692.5	0.381	0.337	0.4	0.391
693	0.381	0.337	0.4	0.391
693.5	0.381	0.337	0.4	0.388
694	0.381	0.337	0.4	0.388
694.5	0.378	0.334	0.403	0.391

695	0.378	0.334	0.403	0.391
695.5	0.378	0.334	0.403	0.391
696	0.378	0.334	0.403	0.391
696.5	0.381	0.334	0.403	0.391
697	0.381	0.334	0.403	0.391
697.5	0.378	0.334	0.403	0.391
698	0.413	0.354	0.442	0.42
698.5	0.447	0.374	0.481	0.452
699	0.481	0.396	0.522	0.483
699.5	0.43	0.369	0.464	0.439
700	0.376	0.339	0.403	0.396
700.5	0.376	0.339	0.403	0.396
701	0.378	0.339	0.403	0.393
701.5	0.378	0.339	0.403	0.393
702	0.378	0.339	0.403	0.393
702.5	0.378	0.339	0.405	0.393
703	0.378	0.339	0.405	0.393
703.5	0.378	0.339	0.405	0.393
704	0.378	0.339	0.405	0.393
704.5	0.376	0.339	0.405	0.393
705	0.376	0.339	0.405	0.393
705.5	0.376	0.337	0.405	0.393
706	0.376	0.337	0.405	0.393
706.5	0.376	0.337	0.405	0.393
707	0.376	0.337	0.405	0.393
707.5	0.376	0.337	0.405	0.393
708	0.378	0.337	0.405	0.393
708.5	0.378	0.337	0.405	0.393
709	0.378	0.337	0.405	0.393
709.5	0.378	0.337	0.405	0.393
710	0.378	0.337	0.405	0.393
710.5	0.378	0.337	0.405	0.393
711	0.378	0.337	0.405	0.393
711.5	0.378	0.337	0.405	0.393

712	0.376	0.337	0.405	0.393
712.5	0.376	0.337	0.405	0.393
713	0.376	0.334	0.405	0.393
713.5	0.376	0.334	0.405	0.393
714	0.376	0.337	0.405	0.393
714.5	0.376	0.337	0.405	0.393
715	0.376	0.337	0.403	0.393
715.5	0.376	0.337	0.403	0.393
716	0.376	0.337	0.403	0.393
716.5	0.376	0.337	0.403	0.393
717	0.376	0.337	0.403	0.391
717.5	0.376	0.337	0.403	0.391
718	0.376	0.337	0.405	0.391
718.5	0.376	0.337	0.405	0.391
719	0.376	0.337	0.405	0.391
719.5	0.376	0.337	0.405	0.391
720	0.376	0.337	0.403	0.391
720.5	0.376	0.337	0.403	0.391
721	0.376	0.334	0.405	0.391
721.5	0.376	0.334	0.405	0.391
722	0.378	0.334	0.403	0.391
722.5	0.378	0.334	0.403	0.391
723	0.376	0.334	0.403	0.391
723.5	0.376	0.334	0.403	0.391
724	0.376	0.334	0.405	0.391
724.5	0.376	0.334	0.405	0.391
725	0.376	0.334	0.403	0.391
725.5	0.376	0.334	0.403	0.391
726	0.376	0.334	0.405	0.391
726.5	0.376	0.337	0.405	0.393
727	0.376	0.337	0.405	0.391
727.5	0.376	0.337	0.405	0.391
728	0.376	0.334	0.405	0.391
728.5	0.376	0.334	0.405	0.391

729	0.376	0.334	0.405	0.391
729.5	0.376	0.337	0.405	0.393
730	0.376	0.337	0.405	0.393
730.5	0.376	0.337	0.405	0.393
731	0.376	0.337	0.405	0.393
731.5	0.376	0.337	0.405	0.393
732	0.376	0.337	0.405	0.393
732.5	0.376	0.334	0.405	0.393
733	0.376	0.334	0.405	0.393
733.5	0.376	0.337	0.403	0.391
734	0.376	0.337	0.403	0.391
734.5	0.376	0.334	0.405	0.393
735	0.376	0.334	0.405	0.393
735.5	0.376	0.334	0.405	0.393
736	0.376	0.334	0.405	0.393
736.5	0.376	0.334	0.405	0.393
737	0.376	0.334	0.405	0.393
737.5	0.376	0.334	0.405	0.391
738	0.376	0.334	0.405	0.391
738.5	0.376	0.334	0.405	0.391
739	0.376	0.334	0.405	0.391
739.5	0.376	0.334	0.405	0.391
740	0.376	0.334	0.405	0.391
740.5	0.376	0.334	0.405	0.391
741	0.376	0.334	0.405	0.391
741.5	0.376	0.334	0.403	0.391
742	0.376	0.334	0.403	0.391
742.5	0.376	0.334	0.403	0.391
743	0.376	0.334	0.403	0.391
743.5	0.376	0.332	0.405	0.391
744	0.376	0.332	0.405	0.391
744.5	0.376	0.334	0.405	0.391
745	0.376	0.334	0.405	0.391
745.5	0.376	0.332	0.405	0.391

746	0.376	0.334	0.405	0.391
746.5	0.376	0.334	0.405	0.391
747	0.376	0.334	0.405	0.391
747.5	0.376	0.334	0.405	0.391
748	0.41	0.354	0.444	0.422
748.5	0.444	0.374	0.486	0.454
749	0.481	0.396	0.527	0.486
749.5	0.43	0.366	0.466	0.439
750	0.376	0.334	0.405	0.391
750.5	0.366	0.322	0.393	0.378
751	0.366	0.322	0.393	0.378
751.5	0.369	0.325	0.396	0.378
752	0.369	0.325	0.396	0.378
752.5	0.369	0.325	0.396	0.378
753	0.369	0.325	0.396	0.378
753.5	0.369	0.325	0.393	0.378
754	0.369	0.325	0.393	0.378
754.5	0.369	0.325	0.393	0.381
755	0.369	0.325	0.393	0.381
755.5	0.369	0.325	0.393	0.381
756	0.369	0.325	0.393	0.381
756.5	0.369	0.325	0.393	0.381
757	0.369	0.325	0.393	0.381
757.5	0.369	0.325	0.393	0.381
758	0.369	0.325	0.393	0.381
758.5	0.371	0.327	0.396	0.381
759	0.371	0.327	0.396	0.383
759.5	0.371	0.327	0.396	0.381
760	0.371	0.327	0.396	0.381
760.5	0.371	0.327	0.396	0.381
761	0.371	0.327	0.396	0.381
761.5	0.371	0.327	0.396	0.381
762	0.371	0.327	0.396	0.381
762.5	0.371	0.327	0.396	0.381

763	0.371	0.327	0.396	0.381
763.5	0.371	0.327	0.396	0.381
764	0.371	0.327	0.396	0.381
764.5	0.371	0.327	0.396	0.381
765	0.371	0.327	0.396	0.381
765.5	0.371	0.327	0.396	0.381
766	0.371	0.327	0.396	0.383
766.5	0.371	0.327	0.396	0.383
767	0.371	0.327	0.398	0.383
767.5	0.371	0.327	0.398	0.383
768	0.371	0.327	0.398	0.383
768.5	0.371	0.327	0.398	0.383
769	0.371	0.327	0.398	0.383
769.5	0.371	0.327	0.398	0.383
770	0.371	0.33	0.398	0.383
770.5	0.371	0.33	0.398	0.383
771	0.371	0.327	0.398	0.383
771.5	0.371	0.327	0.398	0.383
772	0.371	0.33	0.396	0.383
772.5	0.371	0.33	0.396	0.383
773	0.371	0.33	0.398	0.383
773.5	0.371	0.33	0.398	0.383
774	0.371	0.33	0.396	0.383
774.5	0.371	0.33	0.396	0.383
775	0.374	0.33	0.398	0.383
775.5	0.374	0.33	0.398	0.383
776	0.374	0.332	0.398	0.383
776.5	0.374	0.332	0.398	0.383
777	0.374	0.33	0.398	0.386
777.5	0.374	0.33	0.398	0.386
778	0.374	0.33	0.398	0.386
778.5	0.374	0.332	0.398	0.386
779	0.374	0.332	0.398	0.383
779.5	0.374	0.33	0.398	0.383

780	0.374	0.33	0.398	0.383
780.5	0.374	0.332	0.4	0.383
781	0.374	0.332	0.4	0.383
781.5	0.374	0.332	0.4	0.386
782	0.374	0.332	0.4	0.386
782.5	0.374	0.332	0.4	0.386
783	0.374	0.332	0.4	0.386
783.5	0.374	0.332	0.4	0.386
784	0.374	0.332	0.4	0.386
784.5	0.376	0.332	0.4	0.386
785	0.376	0.332	0.4	0.386
785.5	0.376	0.332	0.4	0.386
786	0.376	0.332	0.4	0.386
786.5	0.376	0.332	0.4	0.386
787	0.376	0.332	0.4	0.386
787.5	0.374	0.332	0.4	0.386
788	0.374	0.332	0.4	0.386
788.5	0.376	0.332	0.4	0.386
789	0.376	0.332	0.4	0.386
789.5	0.376	0.332	0.4	0.386
790	0.376	0.332	0.4	0.386
790.5	0.376	0.332	0.4	0.386
791	0.376	0.332	0.4	0.386
791.5	0.376	0.332	0.4	0.386
792	0.376	0.332	0.4	0.386
792.5	0.376	0.334	0.403	0.388
793	0.376	0.334	0.403	0.388
793.5	0.378	0.332	0.403	0.388
794	0.378	0.332	0.403	0.388
794.5	0.378	0.332	0.403	0.388
795	0.378	0.332	0.403	0.388
795.5	0.378	0.332	0.4	0.388
796	0.378	0.332	0.4	0.388
796.5	0.378	0.332	0.4	0.388

797	0.378	0.332	0.403	0.388
797.5	0.378	0.332	0.403	0.388
798	0.378	0.332	0.403	0.388
798.5	0.378	0.332	0.403	0.388
799	0.378	0.332	0.403	0.388
799.5	0.376	0.332	0.403	0.388
800	0.376	0.332	0.403	0.388
800.5	0.376	0.332	0.403	0.388
801	0.376	0.332	0.403	0.388
801.5	0.376	0.332	0.403	0.388
802	0.376	0.332	0.405	0.388
802.5	0.376	0.332	0.405	0.388
803	0.376	0.334	0.405	0.388
803.5	0.376	0.334	0.405	0.388
804	0.376	0.334	0.405	0.388
804.5	0.376	0.334	0.405	0.388
805	0.376	0.332	0.405	0.388
805.5	0.376	0.332	0.405	0.388
806	0.378	0.334	0.403	0.388
806.5	0.378	0.334	0.403	0.388
807	0.378	0.332	0.403	0.388
807.5	0.378	0.332	0.403	0.388
808	0.415	0.354	0.444	0.42
808.5	0.452	0.376	0.486	0.454
809	0.488	0.398	0.53	0.488
809.5	0.435	0.366	0.466	0.439
810	0.378	0.332	0.403	0.388
810.5	0.378	0.332	0.408	0.388
811	0.378	0.332	0.408	0.388
811.5	0.381	0.332	0.408	0.388
812	0.381	0.332	0.408	0.388
812.5	0.381	0.332	0.408	0.388
813	0.378	0.332	0.408	0.391
813.5	0.378	0.332	0.408	0.391

814	0.381	0.332	0.408	0.388
814.5	0.381	0.332	0.408	0.388
815	0.378	0.332	0.41	0.391
815.5	0.378	0.332	0.41	0.391
816	0.378	0.332	0.408	0.391
816.5	0.378	0.332	0.408	0.391
817	0.378	0.332	0.408	0.391
817.5	0.378	0.332	0.408	0.391
818	0.378	0.332	0.408	0.388
818.5	0.378	0.332	0.408	0.388
819	0.378	0.332	0.408	0.391
819.5	0.378	0.332	0.408	0.391
820	0.378	0.334	0.408	0.391
820.5	0.378	0.334	0.408	0.391
821	0.378	0.334	0.408	0.391
821.5	0.378	0.334	0.408	0.391
822	0.378	0.334	0.408	0.391
822.5	0.378	0.334	0.408	0.391
823	0.378	0.334	0.408	0.391
823.5	0.378	0.334	0.408	0.391
824	0.378	0.334	0.408	0.391
824.5	0.378	0.334	0.408	0.391
825	0.378	0.334	0.408	0.391
825.5	0.378	0.334	0.408	0.391
826	0.378	0.334	0.408	0.391
826.5	0.378	0.334	0.408	0.388
827	0.378	0.334	0.408	0.388
827.5	0.378	0.334	0.405	0.391
828	0.378	0.334	0.405	0.391
828.5	0.378	0.334	0.408	0.388
829	0.378	0.334	0.408	0.388
829.5	0.378	0.332	0.405	0.391
830	0.378	0.332	0.405	0.391
830.5	0.381	0.334	0.405	0.391

831	0.381	0.334	0.405	0.391
831.5	0.378	0.334	0.405	0.388
832	0.378	0.334	0.405	0.388
832.5	0.378	0.334	0.405	0.388
833	0.378	0.334	0.405	0.388
833.5	0.378	0.334	0.405	0.388
834	0.378	0.334	0.405	0.388
834.5	0.378	0.332	0.405	0.388
835	0.378	0.332	0.405	0.388
835.5	0.378	0.334	0.405	0.388
836	0.378	0.334	0.405	0.388
836.5	0.378	0.334	0.405	0.388
837	0.378	0.334	0.405	0.388
837.5	0.378	0.334	0.405	0.391
838	0.378	0.334	0.405	0.391
838.5	0.381	0.334	0.405	0.391
839	0.381	0.334	0.405	0.391
839.5	0.381	0.334	0.408	0.391
840	0.381	0.334	0.408	0.391
840.5	0.381	0.334	0.408	0.391
841	0.381	0.334	0.408	0.391
841.5	0.381	0.334	0.405	0.391
842	0.381	0.334	0.408	0.391
842.5	0.381	0.334	0.408	0.391
843	0.378	0.334	0.408	0.391
843.5	0.378	0.334	0.408	0.391
844	0.378	0.332	0.408	0.391
844.5	0.378	0.332	0.408	0.391
845	0.381	0.332	0.408	0.391
845.5	0.381	0.332	0.408	0.391
846	0.378	0.334	0.408	0.391
846.5	0.378	0.334	0.408	0.391
847	0.378	0.334	0.408	0.391
847.5	0.378	0.334	0.408	0.391

848	0.378	0.334	0.408	0.391
848.5	0.378	0.334	0.408	0.391
849	0.378	0.334	0.408	0.393
849.5	0.378	0.334	0.408	0.393
850	0.378	0.334	0.408	0.391
850.5	0.378	0.334	0.408	0.391
851	0.378	0.334	0.408	0.391
851.5	0.378	0.334	0.408	0.391
852	0.378	0.334	0.408	0.393
852.5	0.378	0.334	0.408	0.393
853	0.378	0.332	0.408	0.393
853.5	0.378	0.332	0.408	0.393
854	0.378	0.334	0.408	0.393
854.5	0.378	0.334	0.408	0.393
855	0.378	0.334	0.408	0.393
855.5	0.378	0.334	0.408	0.393
856	0.378	0.334	0.408	0.393
856.5	0.378	0.334	0.408	0.393
857	0.378	0.334	0.408	0.393
857.5	0.378	0.334	0.408	0.393
858	0.378	0.334	0.408	0.393
858.5	0.378	0.334	0.408	0.393
859	0.381	0.334	0.41	0.393
859.5	0.378	0.334	0.41	0.393
860	0.378	0.334	0.408	0.393
860.5	0.381	0.334	0.41	0.393
861	0.381	0.334	0.41	0.393
861.5	0.381	0.334	0.408	0.393
862	0.381	0.334	0.408	0.393
862.5	0.378	0.334	0.408	0.393
863	0.378	0.334	0.408	0.393
863.5	0.381	0.334	0.408	0.393
864	0.381	0.334	0.408	0.393
864.5	0.381	0.334	0.408	0.393

865	0.381	0.334	0.408	0.393
865.5	0.381	0.334	0.408	0.393
866	0.381	0.334	0.408	0.393
866.5	0.381	0.334	0.408	0.393
867	0.381	0.334	0.408	0.393
867.5	0.381	0.334	0.408	0.393
868	0.381	0.334	0.408	0.393
868.5	0.381	0.334	0.408	0.393
869	0.381	0.334	0.408	0.393
869.5	0.381	0.334	0.408	0.393
870	0.381	0.334	0.408	0.393
870.5	0.383	0.337	0.41	0.393
871	0.383	0.337	0.41	0.393
871.5	0.381	0.334	0.408	0.393
872	0.381	0.334	0.408	0.393
872.5	0.381	0.334	0.41	0.393
873	0.381	0.334	0.41	0.393
873.5	0.381	0.334	0.41	0.393
874	0.381	0.334	0.41	0.393
874.5	0.383	0.334	0.41	0.393
875	0.383	0.334	0.41	0.393
875.5	0.383	0.334	0.41	0.393
876	0.383	0.334	0.41	0.393
876.5	0.383	0.334	0.41	0.393
877	0.383	0.334	0.41	0.393
877.5	0.383	0.334	0.41	0.393
878	0.383	0.334	0.41	0.393
878.5	0.383	0.334	0.41	0.393
879	0.383	0.334	0.41	0.393
879.5	0.381	0.334	0.41	0.393
880	0.417	0.356	0.449	0.425
880.5	0.454	0.378	0.488	0.459
881	0.491	0.4	0.53	0.493
881.5	0.437	0.369	0.471	0.444

882	0.383	0.337	0.41	0.393
882.5	0.383	0.325	0.42	0.396
883	0.383	0.325	0.42	0.396
883.5	0.383	0.325	0.417	0.396
884	0.383	0.325	0.417	0.396
884.5	0.381	0.325	0.417	0.396
885	0.381	0.325	0.417	0.396
885.5	0.381	0.325	0.417	0.396
886	0.381	0.325	0.417	0.396
886.5	0.381	0.325	0.417	0.396
887	0.381	0.325	0.417	0.396
887.5	0.381	0.327	0.417	0.396
888	0.381	0.327	0.417	0.396
888.5	0.381	0.327	0.415	0.396
889	0.381	0.327	0.415	0.396
889.5	0.381	0.327	0.415	0.396
890	0.381	0.327	0.415	0.396
890.5	0.381	0.327	0.415	0.396
891	0.381	0.327	0.415	0.396
891.5	0.381	0.327	0.415	0.396
892	0.381	0.327	0.415	0.396
892.5	0.381	0.327	0.415	0.396
893	0.381	0.327	0.415	0.396
893.5	0.381	0.327	0.415	0.393
894	0.381	0.327	0.413	0.393
894.5	0.381	0.327	0.413	0.393
895	0.381	0.33	0.415	0.393
895.5	0.381	0.33	0.415	0.393
896	0.381	0.33	0.413	0.396
896.5	0.381	0.33	0.413	0.396
897	0.381	0.33	0.413	0.393
897.5	0.381	0.33	0.413	0.393
898	0.381	0.33	0.413	0.393
898.5	0.381	0.33	0.413	0.393

899	0.381	0.33	0.413	0.393
899.5	0.381	0.33	0.413	0.393
900	0.381	0.33	0.413	0.393
900.5	0.381	0.33	0.413	0.393
901	0.381	0.33	0.413	0.393
901.5	0.381	0.33	0.413	0.393
902	0.381	0.33	0.413	0.393
902.5	0.381	0.33	0.413	0.393
903	0.381	0.33	0.413	0.393
903.5	0.381	0.33	0.413	0.393
904	0.381	0.33	0.413	0.393
904.5	0.381	0.33	0.413	0.393
905	0.381	0.33	0.41	0.393
905.5	0.381	0.33	0.41	0.393
906	0.381	0.332	0.413	0.393
906.5	0.381	0.332	0.413	0.393
907	0.381	0.332	0.41	0.393
907.5	0.381	0.332	0.41	0.393
908	0.381	0.332	0.41	0.393
908.5	0.381	0.332	0.41	0.393
909	0.381	0.332	0.41	0.393
909.5	0.381	0.332	0.41	0.393
910	0.381	0.332	0.41	0.393
910.5	0.381	0.332	0.41	0.393
911	0.381	0.332	0.41	0.393
911.5	0.381	0.332	0.41	0.393
912	0.381	0.332	0.41	0.391
912.5	0.381	0.332	0.41	0.391
913	0.381	0.332	0.41	0.391
913.5	0.381	0.332	0.41	0.393
914	0.381	0.332	0.41	0.393
914.5	0.381	0.332	0.41	0.393
915	0.381	0.332	0.41	0.393
915.5	0.381	0.332	0.41	0.393

916	0.381	0.332	0.41	0.393
916.5	0.381	0.332	0.41	0.393
917	0.381	0.332	0.41	0.393
917.5	0.381	0.332	0.41	0.391
918	0.381	0.332	0.41	0.391
918.5	0.381	0.332	0.41	0.393
919	0.381	0.332	0.41	0.393
919.5	0.378	0.332	0.41	0.391
920	0.378	0.332	0.41	0.391
920.5	0.381	0.332	0.408	0.393
921	0.381	0.332	0.408	0.393
921.5	0.381	0.332	0.41	0.393
922	0.381	0.332	0.41	0.393
922.5	0.378	0.334	0.41	0.393
923	0.378	0.334	0.41	0.393
923.5	0.378	0.334	0.41	0.393
924	0.378	0.334	0.41	0.393
924.5	0.381	0.334	0.41	0.393
925	0.381	0.334	0.41	0.393
925.5	0.381	0.334	0.41	0.393
926	0.381	0.334	0.41	0.393
926.5	0.381	0.334	0.41	0.393
927	0.381	0.334	0.41	0.393
927.5	0.381	0.334	0.41	0.393
928	0.381	0.334	0.41	0.393
928.5	0.381	0.334	0.41	0.393
929	0.381	0.334	0.41	0.393
929.5	0.381	0.334	0.41	0.393
930	0.381	0.334	0.41	0.393
930.5	0.381	0.334	0.41	0.396
931	0.381	0.334	0.41	0.393
931.5	0.381	0.334	0.41	0.393
932	0.381	0.334	0.41	0.393
932.5	0.381	0.334	0.41	0.393

933	0.381	0.334	0.41	0.393
933.5	0.381	0.334	0.41	0.393
934	0.381	0.334	0.41	0.393
934.5	0.381	0.334	0.41	0.393
935	0.378	0.337	0.408	0.393
935.5	0.378	0.337	0.408	0.393
936	0.381	0.334	0.41	0.393
936.5	0.381	0.334	0.41	0.393
937	0.381	0.337	0.41	0.393
937.5	0.381	0.337	0.41	0.393
938	0.381	0.337	0.41	0.393
938.5	0.381	0.337	0.41	0.393
939	0.381	0.337	0.41	0.393
939.5	0.381	0.337	0.41	0.393
940	0.381	0.334	0.41	0.393
940.5	0.381	0.334	0.41	0.393
941	0.381	0.334	0.41	0.393
941.5	0.381	0.334	0.41	0.393
942	0.381	0.334	0.41	0.396
942.5	0.381	0.334	0.41	0.396
943	0.381	0.337	0.41	0.396
943.5	0.381	0.337	0.41	0.396
944	0.381	0.337	0.41	0.393
944.5	0.381	0.337	0.41	0.393
945	0.381	0.337	0.41	0.393
945.5	0.381	0.337	0.41	0.393
946	0.381	0.334	0.41	0.396
946.5	0.381	0.334	0.41	0.396
947	0.381	0.334	0.41	0.393
947.5	0.381	0.334	0.41	0.393
948	0.381	0.334	0.41	0.396
948.5	0.381	0.334	0.41	0.396
949	0.381	0.334	0.413	0.393
949.5	0.381	0.334	0.413	0.396

950	0.381	0.334	0.41	0.396
950.5	0.381	0.334	0.413	0.396
951	0.381	0.334	0.413	0.396
951.5	0.381	0.337	0.41	0.396
952	0.381	0.337	0.41	0.396
952.5	0.381	0.334	0.41	0.396
953	0.381	0.334	0.41	0.396
953.5	0.381	0.337	0.413	0.396
954	0.381	0.337	0.413	0.396
954.5	0.381	0.337	0.413	0.393
955	0.381	0.337	0.413	0.393
955.5	0.381	0.337	0.413	0.396
956	0.381	0.337	0.413	0.396
956.5	0.381	0.337	0.413	0.396
957	0.381	0.337	0.413	0.396
957.5	0.381	0.337	0.413	0.396
958	0.381	0.337	0.413	0.396
958.5	0.381	0.337	0.413	0.396
959	0.381	0.337	0.413	0.396
959.5	0.381	0.337	0.413	0.396
960	0.381	0.337	0.413	0.396
960.5	0.381	0.337	0.413	0.398
961	0.381	0.337	0.413	0.398
961.5	0.381	0.337	0.413	0.396
962	0.381	0.337	0.413	0.396
962.5	0.381	0.337	0.413	0.396
963	0.381	0.337	0.413	0.396
963.5	0.381	0.337	0.41	0.396
964	0.381	0.337	0.41	0.396
964.5	0.381	0.337	0.413	0.396
965	0.381	0.337	0.413	0.396
965.5	0.381	0.337	0.413	0.396
966	0.417	0.359	0.452	0.43
966.5	0.457	0.381	0.491	0.464

967	0.496	0.405	0.532	0.498
967.5	0.447	0.376	0.476	0.457
968	0.396	0.344	0.42	0.413
968.5	0.396	0.344	0.42	0.413
969	0.396	0.344	0.42	0.41
969.5	0.396	0.344	0.42	0.41
970	0.396	0.342	0.42	0.41
970.5	0.396	0.342	0.42	0.41
971	0.396	0.342	0.42	0.41
971.5	0.396	0.342	0.42	0.41
972	0.393	0.342	0.417	0.41
972.5	0.393	0.342	0.417	0.41
973	0.393	0.342	0.417	0.408
973.5	0.393	0.342	0.417	0.408
974	0.393	0.342	0.417	0.408
974.5	0.393	0.342	0.417	0.408
975	0.393	0.342	0.417	0.408
975.5	0.393	0.342	0.417	0.408
976	0.393	0.342	0.417	0.408
976.5	0.393	0.342	0.417	0.408
977	0.393	0.344	0.415	0.408
977.5	0.393	0.344	0.415	0.408
978	0.393	0.342	0.415	0.408
978.5	0.393	0.344	0.417	0.405
979	0.393	0.342	0.415	0.405
979.5	0.393	0.342	0.415	0.405
980	0.391	0.342	0.415	0.405
980.5	0.393	0.342	0.415	0.405
981	0.393	0.342	0.415	0.405
981.5	0.391	0.342	0.415	0.403
982	0.391	0.342	0.415	0.403
982.5	0.391	0.342	0.415	0.405
983	0.391	0.342	0.415	0.405
983.5	0.391	0.342	0.415	0.403

984	0.391	0.342	0.415	0.403
984.5	0.391	0.342	0.415	0.403
985	0.391	0.342	0.415	0.403
985.5	0.391	0.342	0.415	0.403
986	0.391	0.342	0.415	0.403
986.5	0.391	0.342	0.415	0.403
987	0.391	0.342	0.415	0.403
987.5	0.391	0.342	0.415	0.403
988	0.391	0.342	0.415	0.403
988.5	0.391	0.342	0.415	0.403
989	0.391	0.342	0.415	0.403
989.5	0.388	0.342	0.415	0.403
990	0.388	0.342	0.415	0.403
990.5	0.391	0.342	0.415	0.403
991	0.391	0.342	0.415	0.403
991.5	0.388	0.342	0.415	0.403
992	0.388	0.342	0.415	0.403
992.5	0.391	0.342	0.415	0.403
993	0.391	0.342	0.415	0.403
993.5	0.391	0.342	0.415	0.403
994	0.391	0.342	0.415	0.403
994.5	0.391	0.342	0.415	0.4
995	0.391	0.342	0.415	0.4
995.5	0.388	0.342	0.415	0.403
996	0.388	0.342	0.415	0.403
996.5	0.391	0.342	0.413	0.4
997	0.388	0.344	0.413	0.4
997.5	0.388	0.342	0.413	0.4
998	0.388	0.342	0.413	0.403
998.5	0.388	0.342	0.415	0.403
999	0.388	0.342	0.415	0.403
999.5	0.388	0.342	0.415	0.403
1000	0.388	0.342	0.415	0.403
1000.5	0.388	0.342	0.415	0.403

1001	0.388	0.342	0.413	0.4
1001.5	0.388	0.342	0.413	0.4
1002	0.391	0.342	0.415	0.4
1002.5	0.391	0.342	0.415	0.4
1003	0.388	0.342	0.413	0.4
1003.5	0.388	0.342	0.413	0.4
1004	0.388	0.342	0.413	0.4
1004.5	0.388	0.342	0.413	0.4
1005	0.388	0.342	0.413	0.4
1005.5	0.388	0.342	0.413	0.4
1006	0.388	0.339	0.413	0.4
1006.5	0.388	0.339	0.413	0.4
1007	0.388	0.342	0.413	0.4
1007.5	0.388	0.342	0.413	0.4
1008	0.388	0.342	0.413	0.4
1008.5	0.388	0.342	0.413	0.4
1009	0.388	0.339	0.413	0.398
1009.5	0.388	0.339	0.413	0.398
1010	0.388	0.339	0.413	0.398
1010.5	0.388	0.339	0.413	0.398
1011	0.386	0.339	0.413	0.398
1011.5	0.386	0.339	0.413	0.398
1012	0.386	0.339	0.413	0.398
1012.5	0.386	0.339	0.413	0.398
1013	0.388	0.339	0.413	0.398
1013.5	0.388	0.339	0.413	0.398
1014	0.388	0.339	0.413	0.398
1014.5	0.388	0.339	0.413	0.398
1015	0.386	0.339	0.413	0.398
1015.5	0.386	0.339	0.413	0.398
1016	0.386	0.339	0.413	0.398
1016.5	0.386	0.339	0.413	0.398
1017	0.386	0.339	0.413	0.398
1017.5	0.386	0.339	0.413	0.398

1018	0.386	0.339	0.413	0.398
1018.5	0.386	0.339	0.413	0.398
1019	0.386	0.339	0.413	0.398
1019.5	0.386	0.339	0.413	0.398
1020	0.386	0.339	0.413	0.398
1020.5	0.386	0.339	0.413	0.398
1021	0.386	0.339	0.413	0.398
1021.5	0.386	0.339	0.41	0.398
1022	0.386	0.339	0.41	0.398
1022.5	0.386	0.339	0.413	0.398
1023	0.386	0.339	0.413	0.398
1023.5	0.386	0.339	0.413	0.398
1024	0.386	0.339	0.413	0.398
1024.5	0.386	0.339	0.413	0.398
1025	0.386	0.339	0.413	0.398
1025.5	0.386	0.339	0.413	0.398
1026	0.386	0.339	0.413	0.398
1026.5	0.386	0.339	0.413	0.398
1027	0.386	0.339	0.413	0.398
1027.5	0.386	0.339	0.413	0.396
1028	0.386	0.339	0.413	0.396
1028.5	0.386	0.339	0.413	0.398
1029	0.386	0.339	0.413	0.398
1029.5	0.386	0.339	0.413	0.398
1030	0.386	0.339	0.413	0.398
1030.5	0.386	0.339	0.413	0.398
1031	0.386	0.339	0.413	0.398
1031.5	0.386	0.339	0.41	0.398
1032	0.386	0.339	0.41	0.398
1032.5	0.386	0.339	0.413	0.396
1033	0.386	0.339	0.413	0.396
1033.5	0.386	0.339	0.413	0.396
1034	0.386	0.339	0.413	0.396
1034.5	0.386	0.339	0.413	0.398

1035	0.386	0.342	0.413	0.398
1035.5	0.386	0.339	0.413	0.398
1036	0.386	0.339	0.413	0.398
1036.5	0.386	0.339	0.413	0.398
1037	0.386	0.339	0.413	0.398
1037.5	0.386	0.339	0.413	0.398
1038	0.386	0.339	0.413	0.398
1038.5	0.386	0.339	0.413	0.398
1039	0.386	0.339	0.413	0.398
1039.5	0.386	0.339	0.413	0.398
1040	0.386	0.339	0.413	0.398
1040.5	0.386	0.339	0.413	0.398
1041	0.386	0.339	0.413	0.398
1041.5	0.386	0.339	0.413	0.398
1042	0.386	0.339	0.413	0.398
1042.5	0.386	0.339	0.413	0.398
1043	0.386	0.339	0.413	0.398
1043.5	0.386	0.339	0.413	0.398
1044	0.386	0.339	0.413	0.398
1044.5	0.386	0.339	0.413	0.398
1045	0.386	0.339	0.413	0.398
1045.5	0.386	0.339	0.413	0.398
1046	0.386	0.339	0.413	0.398
1046.5	0.386	0.339	0.413	0.398
1047	0.383	0.339	0.413	0.398
1047.5	0.383	0.339	0.413	0.398
1048	0.386	0.339	0.413	0.398
1048.5	0.386	0.339	0.413	0.398
1049	0.386	0.339	0.413	0.398
1049.5	0.386	0.339	0.413	0.398
1050	0.386	0.339	0.413	0.398
1050.5	0.386	0.339	0.413	0.398
1051	0.386	0.339	0.413	0.398
1051.5	0.386	0.339	0.413	0.398

1052	0.386	0.339	0.413	0.398
1052.5	0.386	0.339	0.413	0.398
1053	0.386	0.339	0.413	0.398
1053.5	0.386	0.339	0.413	0.398
1054	0.386	0.337	0.413	0.398
1054.5	0.386	0.339	0.413	0.398
1055	0.386	0.339	0.413	0.398
1055.5	0.386	0.339	0.413	0.398
1056	0.386	0.339	0.413	0.398
1056.5	0.386	0.339	0.413	0.398
1057	0.386	0.339	0.413	0.398
1057.5	0.386	0.339	0.413	0.398
1058	0.386	0.339	0.413	0.398
1058.5	0.386	0.339	0.413	0.398
1059	0.386	0.339	0.413	0.398
1059.5	0.386	0.339	0.413	0.398
1060	0.386	0.339	0.413	0.398
1060.5	0.386	0.339	0.413	0.398
1061	0.386	0.339	0.413	0.398
1061.5	0.386	0.339	0.41	0.398
1062	0.386	0.339	0.41	0.398
1062.5	0.386	0.339	0.413	0.398
1063	0.386	0.339	0.413	0.398
1063.5	0.386	0.339	0.413	0.398
1064	0.386	0.339	0.413	0.398
1064.5	0.386	0.339	0.41	0.398
1065	0.386	0.339	0.41	0.398
1065.5	0.386	0.339	0.413	0.398
1066	0.386	0.339	0.413	0.398
1066.5	0.386	0.339	0.413	0.396
1067	0.386	0.339	0.413	0.396
1067.5	0.386	0.339	0.41	0.398
1068	0.386	0.339	0.41	0.398
1068.5	0.386	0.339	0.41	0.396

1069	0.422	0.364	0.452	0.43
1069.5	0.461	0.388	0.493	0.464
1070	0.5	0.413	0.537	0.5
1070.5	0.452	0.386	0.479	0.457
1071	0.4	0.356	0.42	0.41
1071.5	0.4	0.356	0.42	0.41
1072	0.4	0.354	0.42	0.408
1072.5	0.4	0.354	0.42	0.408
1073	0.4	0.354	0.42	0.408
1073.5	0.398	0.354	0.42	0.408
1074	0.398	0.354	0.42	0.408
1074.5	0.398	0.354	0.42	0.408
1075	0.398	0.354	0.42	0.408
1075.5	0.398	0.354	0.42	0.408
1076	0.398	0.354	0.42	0.408
1076.5	0.398	0.354	0.417	0.408
1077	0.398	0.354	0.417	0.408
1077.5	0.398	0.354	0.417	0.408
1078	0.398	0.354	0.417	0.408
1078.5	0.398	0.352	0.417	0.405
1079	0.398	0.352	0.417	0.405
1079.5	0.396	0.352	0.417	0.405
1080	0.396	0.352	0.417	0.405
1080.5	0.396	0.352	0.417	0.405
1081	0.396	0.352	0.417	0.405
1081.5	0.396	0.352	0.417	0.405
1082	0.396	0.349	0.417	0.405
1082.5	0.396	0.349	0.417	0.405
1083	0.396	0.349	0.415	0.405
1083.5	0.396	0.349	0.415	0.405
1084	0.393	0.349	0.415	0.403
1084.5	0.393	0.349	0.415	0.403
1085	0.393	0.349	0.415	0.403
1085.5	0.393	0.349	0.415	0.403

1086	0.393	0.349	0.415	0.403
1086.5	0.393	0.349	0.415	0.403
1087	0.393	0.349	0.415	0.403
1087.5	0.393	0.349	0.415	0.403
1088	0.393	0.349	0.415	0.403
1088.5	0.393	0.349	0.415	0.403
1089	0.393	0.349	0.415	0.403
1089.5	0.393	0.349	0.415	0.403
1090	0.391	0.349	0.415	0.403
1090.5	0.391	0.349	0.415	0.403
1091	0.391	0.349	0.415	0.403
1091.5	0.391	0.349	0.415	0.403
1092	0.391	0.349	0.415	0.4
1092.5	0.391	0.349	0.415	0.4
1093	0.391	0.349	0.415	0.403
1093.5	0.391	0.349	0.415	0.403
1094	0.391	0.347	0.415	0.4
1094.5	0.391	0.347	0.415	0.4
1095	0.391	0.347	0.413	0.4
1095.5	0.391	0.347	0.413	0.4
1096	0.391	0.347	0.415	0.4
1096.5	0.391	0.347	0.415	0.4
1097	0.388	0.347	0.413	0.403
1097.5	0.391	0.347	0.413	0.4
1098	0.391	0.347	0.413	0.4
1098.5	0.391	0.347	0.413	0.4
1099	0.388	0.347	0.413	0.4
1099.5	0.388	0.347	0.413	0.403
1100	0.388	0.347	0.413	0.403
1100.5	0.388	0.347	0.413	0.4
1101	0.388	0.347	0.413	0.4
1101.5	0.388	0.347	0.413	0.4
1102	0.388	0.347	0.413	0.4
1102.5	0.388	0.347	0.413	0.4

1103	0.388	0.347	0.413	0.4
1103.5	0.388	0.347	0.413	0.4
1104	0.388	0.347	0.413	0.4
1104.5	0.388	0.347	0.413	0.4
1105	0.388	0.347	0.413	0.4
1105.5	0.388	0.347	0.413	0.4
1106	0.388	0.347	0.413	0.4
1106.5	0.388	0.347	0.413	0.4
1107	0.388	0.347	0.413	0.4
1107.5	0.388	0.347	0.413	0.4
1108	0.388	0.347	0.413	0.4
1108.5	0.388	0.344	0.413	0.4
1109	0.388	0.344	0.413	0.4
1109.5	0.388	0.347	0.413	0.4
1110	0.388	0.347	0.413	0.4
1110.5	0.388	0.344	0.413	0.4
1111	0.388	0.344	0.413	0.4
1111.5	0.388	0.344	0.413	0.4
1112	0.388	0.344	0.413	0.4
1112.5	0.388	0.344	0.413	0.4
1113	0.388	0.344	0.413	0.4
1113.5	0.386	0.344	0.413	0.4
1114	0.386	0.344	0.413	0.4
1114.5	0.386	0.344	0.413	0.4
1115	0.386	0.344	0.413	0.4
1115.5	0.386	0.344	0.413	0.398
1116	0.386	0.344	0.413	0.398
1116.5	0.386	0.344	0.413	0.4
1117	0.386	0.344	0.413	0.4
1117.5	0.386	0.344	0.413	0.4
1118	0.386	0.344	0.413	0.398
1118.5	0.386	0.344	0.413	0.398
1119	0.386	0.344	0.413	0.4
1119.5	0.386	0.344	0.413	0.4

1120	0.386	0.344	0.413	0.4
1120.5	0.386	0.344	0.413	0.4
1121	0.386	0.344	0.413	0.4
1121.5	0.386	0.344	0.413	0.4
1122	0.386	0.344	0.413	0.398
1122.5	0.386	0.344	0.413	0.398
1123	0.386	0.344	0.413	0.4
1123.5	0.386	0.344	0.413	0.4
1124	0.386	0.344	0.413	0.4
1124.5	0.386	0.344	0.413	0.4
1125	0.386	0.344	0.413	0.4
1125.5	0.386	0.344	0.413	0.4
1126	0.386	0.344	0.413	0.4
1126.5	0.386	0.344	0.413	0.4
1127	0.386	0.344	0.413	0.4
1127.5	0.386	0.344	0.413	0.4
1128	0.386	0.344	0.413	0.4
1128.5	0.386	0.344	0.413	0.4
1129	0.386	0.342	0.413	0.4
1129.5	0.386	0.342	0.413	0.4
1130	0.386	0.344	0.413	0.4
1130.5	0.386	0.344	0.413	0.4
1131	0.386	0.344	0.413	0.4
1131.5	0.386	0.344	0.413	0.4
1132	0.386	0.342	0.413	0.398
1132.5	0.386	0.342	0.413	0.398
1133	0.386	0.342	0.413	0.4
1133.5	0.386	0.342	0.413	0.4
1134	0.386	0.344	0.413	0.398
1134.5	0.386	0.344	0.413	0.398
1135	0.386	0.342	0.41	0.398
1135.5	0.386	0.344	0.41	0.398
1136	0.386	0.342	0.41	0.398
1136.5	0.386	0.344	0.41	0.4

1137	0.386	0.344	0.41	0.398
1137.5	0.386	0.342	0.41	0.398
1138	0.386	0.342	0.41	0.398
1138.5	0.386	0.342	0.413	0.4
1139	0.386	0.342	0.413	0.4
1139.5	0.386	0.344	0.413	0.4
1140	0.386	0.344	0.413	0.4
1140.5	0.386	0.344	0.413	0.4
1141	0.386	0.344	0.413	0.4
1141.5	0.386	0.342	0.413	0.398
1142	0.386	0.342	0.413	0.398
1142.5	0.386	0.342	0.413	0.398
1143	0.386	0.342	0.413	0.398
1143.5	0.386	0.342	0.413	0.398
1144	0.386	0.342	0.413	0.398
1144.5	0.386	0.342	0.413	0.398
1145	0.386	0.342	0.413	0.398
1145.5	0.386	0.342	0.413	0.398
1146	0.386	0.342	0.413	0.398
1146.5	0.386	0.344	0.413	0.398
1147	0.386	0.344	0.413	0.398
1147.5	0.386	0.342	0.413	0.398
1148	0.386	0.342	0.413	0.398
1148.5	0.386	0.342	0.413	0.398
1149	0.386	0.342	0.413	0.398
1149.5	0.386	0.342	0.413	0.398
1150	0.386	0.342	0.413	0.398
1150.5	0.386	0.342	0.41	0.398
1151	0.386	0.342	0.41	0.398
1151.5	0.386	0.342	0.41	0.398
1152	0.386	0.342	0.41	0.398
1152.5	0.386	0.342	0.41	0.4
1153	0.386	0.342	0.41	0.4
1153.5	0.386	0.342	0.41	0.398

1154	0.386	0.342	0.41	0.398
1154.5	0.386	0.342	0.41	0.398
1155	0.386	0.342	0.41	0.398
1155.5	0.388	0.342	0.41	0.398
1156	0.386	0.342	0.41	0.398
1156.5	0.386	0.339	0.41	0.398
1157	0.388	0.342	0.41	0.398
1157.5	0.388	0.342	0.41	0.398
1158	0.386	0.342	0.41	0.398
1158.5	0.386	0.342	0.41	0.398
1159	0.386	0.339	0.41	0.398
1159.5	0.386	0.339	0.41	0.398
1160	0.386	0.339	0.41	0.398
1160.5	0.386	0.339	0.41	0.398
1161	0.386	0.339	0.41	0.398
1161.5	0.386	0.339	0.41	0.398
1162	0.386	0.339	0.41	0.398
1162.5	0.386	0.339	0.41	0.398
1163	0.386	0.339	0.41	0.398
1163.5	0.386	0.339	0.41	0.398
1164	0.386	0.339	0.41	0.398
1164.5	0.386	0.339	0.41	0.398
1165	0.386	0.339	0.41	0.398
1165.5	0.386	0.339	0.41	0.398
1166	0.383	0.339	0.41	0.398
1166.5	0.383	0.339	0.41	0.398
1167	0.383	0.339	0.41	0.398
1167.5	0.383	0.339	0.41	0.398
1168	0.383	0.339	0.413	0.398
1168.5	0.383	0.339	0.413	0.398
1169	0.383	0.342	0.41	0.398
1169.5	0.383	0.342	0.41	0.398
1170	0.386	0.342	0.413	0.398
1170.5	0.386	0.342	0.413	0.398

1171	0.386	0.342	0.413	0.4
1171.5	0.386	0.342	0.413	0.4
1172	0.386	0.342	0.413	0.398
1172.5	0.386	0.342	0.413	0.398
1173	0.386	0.342	0.413	0.398
1173.5	0.386	0.342	0.413	0.398
1174	0.386	0.342	0.413	0.398
1174.5	0.386	0.342	0.413	0.398
1175	0.386	0.342	0.413	0.398
1175.5	0.386	0.342	0.413	0.398
1176	0.386	0.342	0.413	0.4
1176.5	0.386	0.342	0.413	0.4
1177	0.386	0.342	0.413	0.4
1177.5	0.386	0.342	0.413	0.398
1178	0.386	0.342	0.413	0.398
1178.5	0.386	0.342	0.413	0.398
1179	0.386	0.342	0.413	0.398
1179.5	0.386	0.342	0.41	0.4
1180	0.386	0.342	0.41	0.4
1180.5	0.386	0.339	0.413	0.398
1181	0.386	0.339	0.413	0.398
1181.5	0.388	0.342	0.41	0.398
1182	0.388	0.342	0.41	0.398
1182.5	0.388	0.342	0.41	0.398
1183	0.388	0.342	0.41	0.398
1183.5	0.386	0.342	0.413	0.398
1184	0.386	0.342	0.413	0.398
1184.5	0.386	0.342	0.413	0.398
1185	0.386	0.342	0.413	0.398
1185.5	0.386	0.342	0.41	0.398
1186	0.386	0.342	0.41	0.398
1186.5	0.388	0.342	0.41	0.398
1187	0.388	0.342	0.41	0.398
1187.5	0.386	0.342	0.41	0.398

1188	0.386	0.342	0.41	0.398
1188.5	0.386	0.339	0.41	0.398
1189	0.386	0.339	0.41	0.398
1189.5	0.386	0.339	0.41	0.398
1190	0.386	0.339	0.41	0.398
1190.5	0.386	0.339	0.41	0.398
1191	0.386	0.339	0.41	0.398
1191.5	0.386	0.339	0.413	0.398
1192	0.386	0.339	0.413	0.398
1192.5	0.386	0.339	0.413	0.398
1193	0.386	0.339	0.41	0.398
1193.5	0.425	0.364	0.454	0.435
1194	0.466	0.391	0.498	0.471
1194.5	0.508	0.417	0.542	0.508
1195	0.447	0.378	0.476	0.454
1195.5	0.386	0.339	0.41	0.398
1196	0.396	0.352	0.422	0.388
1196.5	0.396	0.352	0.422	0.388
1197	0.396	0.352	0.42	0.388
1197.5	0.396	0.352	0.42	0.388
1198	0.396	0.352	0.42	0.386
1198.5	0.396	0.352	0.417	0.388
1199	0.396	0.352	0.417	0.388
1199.5	0.393	0.352	0.417	0.388
1200	0.393	0.352	0.417	0.388
1200.5	0.393	0.352	0.42	0.388
1201	0.393	0.352	0.42	0.388
1201.5	0.393	0.352	0.417	0.388
1202	0.393	0.352	0.417	0.388
1202.5	0.393	0.349	0.417	0.388
1203	0.393	0.349	0.417	0.388
1203.5	0.393	0.349	0.417	0.388
1204	0.393	0.349	0.417	0.388
1204.5	0.393	0.349	0.417	0.388

1205	0.393	0.349	0.417	0.388
1205.5	0.391	0.349	0.417	0.388
1206	0.391	0.349	0.417	0.388
1206.5	0.391	0.349	0.417	0.388
1207	0.391	0.349	0.417	0.388
1207.5	0.393	0.349	0.417	0.391
1208	0.393	0.349	0.417	0.391
1208.5	0.391	0.349	0.417	0.391
1209	0.391	0.349	0.417	0.391
1209.5	0.391	0.349	0.415	0.391
1210	0.391	0.349	0.415	0.391
1210.5	0.391	0.349	0.417	0.391
1211	0.391	0.349	0.417	0.391
1211.5	0.391	0.349	0.417	0.391
1212	0.391	0.349	0.417	0.391
1212.5	0.391	0.349	0.417	0.391
1213	0.391	0.349	0.417	0.391
1213.5	0.391	0.347	0.415	0.393
1214	0.391	0.347	0.415	0.393
1214.5	0.391	0.347	0.415	0.393
1215	0.391	0.347	0.415	0.393
1215.5	0.391	0.347	0.415	0.393
1216	0.391	0.347	0.415	0.393
1216.5	0.391	0.347	0.415	0.393
1217	0.391	0.349	0.417	0.393
1217.5	0.391	0.347	0.415	0.393
1218	0.391	0.347	0.415	0.393
1218.5	0.391	0.347	0.415	0.393
1219	0.391	0.347	0.415	0.393
1219.5	0.391	0.347	0.415	0.393
1220	0.391	0.347	0.415	0.393
1220.5	0.391	0.347	0.415	0.393
1221	0.391	0.347	0.415	0.393
1221.5	0.391	0.347	0.415	0.393

1222	0.391	0.347	0.415	0.393
1222.5	0.391	0.347	0.415	0.393
1223	0.388	0.347	0.415	0.396
1223.5	0.388	0.347	0.415	0.396
1224	0.388	0.347	0.415	0.396
1224.5	0.388	0.347	0.415	0.396
1225	0.388	0.347	0.415	0.393
1225.5	0.388	0.347	0.415	0.393
1226	0.388	0.347	0.417	0.396
1226.5	0.388	0.347	0.417	0.396
1227	0.388	0.347	0.415	0.396
1227.5	0.388	0.347	0.415	0.396
1228	0.388	0.347	0.415	0.396
1228.5	0.388	0.347	0.415	0.396
1229	0.388	0.347	0.415	0.396
1229.5	0.388	0.347	0.415	0.396
1230	0.388	0.347	0.415	0.396
1230.5	0.388	0.347	0.415	0.396
1231	0.388	0.347	0.415	0.396
1231.5	0.388	0.347	0.415	0.396
1232	0.388	0.347	0.415	0.396
1232.5	0.388	0.347	0.415	0.396
1233	0.388	0.347	0.415	0.396
1233.5	0.388	0.347	0.415	0.396
1234	0.388	0.344	0.415	0.396
1234.5	0.388	0.344	0.415	0.396
1235	0.388	0.347	0.415	0.396
1235.5	0.388	0.344	0.415	0.396
1236	0.388	0.344	0.415	0.396
1236.5	0.388	0.344	0.413	0.396
1237	0.388	0.344	0.413	0.396
1237.5	0.388	0.347	0.413	0.396
1238	0.388	0.347	0.413	0.396
1238.5	0.388	0.347	0.413	0.396

1239	0.388	0.347	0.413	0.396
1239.5	0.388	0.347	0.413	0.396
1240	0.388	0.347	0.413	0.396
1240.5	0.388	0.347	0.413	0.398
1241	0.388	0.347	0.413	0.398
1241.5	0.388	0.347	0.415	0.396
1242	0.388	0.347	0.415	0.396
1242.5	0.388	0.344	0.413	0.396
1243	0.388	0.344	0.413	0.396
1243.5	0.388	0.347	0.413	0.396
1244	0.388	0.347	0.413	0.396
1244.5	0.388	0.344	0.413	0.398
1245	0.388	0.344	0.413	0.398
1245.5	0.388	0.347	0.415	0.398
1246	0.388	0.347	0.415	0.398
1246.5	0.388	0.344	0.413	0.398
1247	0.388	0.344	0.413	0.398
1247.5	0.388	0.347	0.413	0.398
1248	0.388	0.347	0.413	0.398
1248.5	0.388	0.344	0.413	0.398
1249	0.388	0.344	0.413	0.398
1249.5	0.388	0.344	0.413	0.398
1250	0.388	0.344	0.413	0.398
1250.5	0.388	0.344	0.413	0.398
1251	0.388	0.344	0.413	0.398
1251.5	0.388	0.344	0.413	0.396
1252	0.388	0.344	0.413	0.396
1252.5	0.386	0.344	0.413	0.396
1253	0.386	0.344	0.413	0.396
1253.5	0.388	0.344	0.413	0.398
1254	0.386	0.344	0.413	0.398
1254.5	0.386	0.344	0.413	0.396
1255	0.386	0.344	0.413	0.396
1255.5	0.386	0.344	0.413	0.398

1256	0.386	0.344	0.413	0.398
1256.5	0.386	0.344	0.413	0.398
1257	0.386	0.344	0.413	0.398
1257.5	0.386	0.344	0.413	0.398
1258	0.386	0.344	0.413	0.396
1258.5	0.386	0.344	0.413	0.396
1259	0.386	0.344	0.413	0.398
1259.5	0.386	0.344	0.413	0.398
1260	0.388	0.344	0.413	0.396
1260.5	0.388	0.344	0.413	0.396
1261	0.386	0.344	0.413	0.398
1261.5	0.386	0.344	0.413	0.398
1262	0.388	0.344	0.413	0.398
1262.5	0.388	0.344	0.413	0.398
1263	0.386	0.344	0.41	0.398
1263.5	0.386	0.344	0.41	0.398
1264	0.386	0.344	0.413	0.398
1264.5	0.386	0.344	0.413	0.398
1265	0.388	0.344	0.413	0.398
1265.5	0.388	0.344	0.413	0.398
1266	0.388	0.344	0.41	0.398
1266.5	0.388	0.344	0.41	0.398
1267	0.386	0.344	0.41	0.398
1267.5	0.386	0.344	0.41	0.398
1268	0.386	0.344	0.413	0.398
1268.5	0.386	0.344	0.413	0.398
1269	0.386	0.344	0.413	0.398
1269.5	0.386	0.344	0.413	0.398
1270	0.386	0.344	0.413	0.398
1270.5	0.386	0.344	0.413	0.398
1271	0.386	0.344	0.41	0.398
1271.5	0.386	0.344	0.41	0.398
1272	0.386	0.344	0.413	0.398
1272.5	0.386	0.344	0.413	0.398

1273	0.386	0.344	0.413	0.398
1273.5	0.386	0.344	0.413	0.398
1274	0.386	0.344	0.413	0.398
1274.5	0.386	0.344	0.413	0.398
1275	0.386	0.344	0.413	0.398
1275.5	0.386	0.344	0.413	0.398
1276	0.386	0.344	0.413	0.398
1276.5	0.386	0.344	0.413	0.4
1277	0.386	0.344	0.413	0.4
1277.5	0.386	0.344	0.413	0.4
1278	0.386	0.344	0.413	0.4
1278.5	0.386	0.344	0.413	0.4
1279	0.386	0.344	0.413	0.4
1279.5	0.386	0.344	0.413	0.4
1280	0.386	0.344	0.413	0.4
1280.5	0.386	0.344	0.413	0.4
1281	0.386	0.344	0.413	0.4
1281.5	0.386	0.344	0.413	0.4
1282	0.386	0.344	0.413	0.4
1282.5	0.386	0.344	0.413	0.4
1283	0.386	0.344	0.413	0.4
1283.5	0.388	0.344	0.413	0.4
1284	0.388	0.344	0.413	0.4
1284.5	0.388	0.344	0.413	0.4
1285	0.388	0.344	0.413	0.4
1285.5	0.388	0.344	0.413	0.4
1286	0.388	0.344	0.413	0.4
1286.5	0.388	0.344	0.413	0.4
1287	0.388	0.344	0.413	0.4
1287.5	0.386	0.344	0.413	0.4
1288	0.386	0.344	0.413	0.4
1288.5	0.386	0.344	0.413	0.4
1289	0.386	0.344	0.413	0.4
1289.5	0.386	0.344	0.413	0.4

1290	0.386	0.344	0.413	0.4
1290.5	0.386	0.344	0.413	0.4
1291	0.386	0.344	0.413	0.4
1291.5	0.388	0.344	0.413	0.4
1292	0.388	0.344	0.413	0.4
1292.5	0.388	0.344	0.413	0.4
1293	0.388	0.344	0.413	0.4
1293.5	0.388	0.344	0.413	0.4
1294	0.388	0.344	0.413	0.4
1294.5	0.388	0.344	0.413	0.4
1295	0.388	0.344	0.413	0.4
1295.5	0.388	0.344	0.413	0.4
1296	0.388	0.344	0.413	0.4
1296.5	0.388	0.344	0.413	0.4
1297	0.388	0.347	0.415	0.4
1297.5	0.388	0.347	0.415	0.4
1298	0.388	0.347	0.413	0.4
1298.5	0.388	0.347	0.413	0.4
1299	0.388	0.344	0.415	0.4
1299.5	0.388	0.344	0.415	0.4
1300	0.388	0.344	0.413	0.4
1300.5	0.388	0.344	0.413	0.4
1301	0.386	0.344	0.413	0.4
1301.5	0.386	0.344	0.413	0.4
1302	0.386	0.344	0.413	0.4
1302.5	0.386	0.344	0.413	0.4
1303	0.386	0.347	0.413	0.4
1303.5	0.386	0.347	0.413	0.4
1304	0.386	0.347	0.413	0.4
1304.5	0.386	0.347	0.413	0.4
1305	0.388	0.347	0.415	0.4
1305.5	0.388	0.347	0.415	0.4
1306	0.388	0.347	0.415	0.4
1306.5	0.388	0.347	0.415	0.4

1307	0.386	0.347	0.415	0.4
1307.5	0.386	0.347	0.415	0.4
1308	0.386	0.347	0.415	0.4
1308.5	0.386	0.347	0.415	0.4
1309	0.388	0.347	0.413	0.4
1309.5	0.388	0.347	0.413	0.4
1310	0.388	0.347	0.413	0.4
1310.5	0.388	0.347	0.413	0.4
1311	0.388	0.347	0.413	0.4
1311.5	0.388	0.347	0.415	0.4
1312	0.388	0.347	0.415	0.4
1312.5	0.388	0.347	0.415	0.4
1313	0.388	0.347	0.415	0.4
1313.5	0.388	0.347	0.413	0.4
1314	0.388	0.347	0.413	0.4
1314.5	0.388	0.347	0.413	0.4
1315	0.388	0.347	0.413	0.4
1315.5	0.388	0.347	0.413	0.4
1316	0.388	0.347	0.413	0.4
1316.5	0.386	0.347	0.413	0.4
1317	0.386	0.347	0.413	0.4
1317.5	0.388	0.347	0.415	0.4
1318	0.388	0.347	0.415	0.4
1318.5	0.386	0.347	0.413	0.4
1319	0.386	0.347	0.413	0.4
1319.5	0.388	0.347	0.413	0.4
1320	0.388	0.347	0.413	0.4
1320.5	0.388	0.347	0.413	0.4
1321	0.388	0.347	0.413	0.4
1321.5	0.386	0.347	0.413	0.4
1322	0.386	0.347	0.413	0.4
1322.5	0.388	0.347	0.413	0.4
1323	0.388	0.347	0.413	0.4
1323.5	0.388	0.347	0.415	0.4

1324	0.388	0.347	0.415	0.4
1324.5	0.388	0.344	0.413	0.403
1325	0.388	0.344	0.413	0.403
1325.5	0.388	0.344	0.413	0.4
1326	0.388	0.344	0.413	0.4
1326.5	0.388	0.344	0.413	0.4
1327	0.388	0.344	0.413	0.4
1327.5	0.388	0.347	0.413	0.4
1328	0.388	0.347	0.413	0.4
1328.5	0.388	0.347	0.413	0.4
1329	0.388	0.347	0.413	0.4
1329.5	0.388	0.347	0.413	0.4
1330	0.388	0.347	0.413	0.4
1330.5	0.388	0.347	0.415	0.4
1331	0.386	0.344	0.413	0.4
1331.5	0.386	0.344	0.413	0.4
1332	0.386	0.344	0.413	0.4
1332.5	0.386	0.344	0.413	0.4
1333	0.386	0.344	0.413	0.4
1333.5	0.386	0.344	0.413	0.4
1334	0.388	0.344	0.413	0.4
1334.5	0.388	0.344	0.413	0.4
1335	0.386	0.344	0.413	0.4
1335.5	0.386	0.344	0.413	0.4
1336	0.386	0.347	0.413	0.4
1336.5	0.386	0.347	0.413	0.4
1337	0.388	0.344	0.413	0.403
1337.5	0.388	0.344	0.413	0.403
1338	0.388	0.347	0.413	0.403
1338.5	0.388	0.347	0.413	0.403
1339	0.386	0.347	0.413	0.403
1339.5	0.386	0.347	0.413	0.403
1340	0.388	0.344	0.413	0.403
1340.5	0.388	0.344	0.413	0.403

1341	0.386	0.344	0.415	0.403
1341.5	0.386	0.344	0.415	0.403
1342	0.427	0.371	0.457	0.439
1342.5	0.469	0.398	0.5	0.476
1343	0.513	0.425	0.544	0.515
1343.5	0.452	0.388	0.479	0.461
1344	0.388	0.349	0.413	0.408
1344.5	0.391	0.352	0.415	0.405
1345	0.393	0.356	0.417	0.403
1345.5	0.393	0.356	0.415	0.403
1346	0.393	0.356	0.415	0.403
1346.5	0.393	0.354	0.415	0.403
1347	0.393	0.354	0.415	0.403
1347.5	0.393	0.354	0.415	0.4
1348	0.393	0.354	0.415	0.4
1348.5	0.393	0.354	0.417	0.4
1349	0.393	0.354	0.417	0.4
1349.5	0.393	0.354	0.415	0.4
1350	0.393	0.354	0.415	0.4
1350.5	0.393	0.354	0.415	0.4
1351	0.393	0.354	0.415	0.4
1351.5	0.391	0.354	0.415	0.4
1352	0.391	0.354	0.415	0.4
1352.5	0.391	0.354	0.417	0.4
1353	0.391	0.354	0.417	0.4
1353.5	0.391	0.352	0.415	0.4
1354	0.391	0.352	0.415	0.4
1354.5	0.391	0.352	0.415	0.4
1355	0.391	0.352	0.415	0.4
1355.5	0.391	0.352	0.415	0.4
1356	0.391	0.352	0.415	0.4
1356.5	0.391	0.352	0.413	0.4
1357	0.391	0.352	0.413	0.4
1357.5	0.391	0.352	0.415	0.4

1358	0.391	0.352	0.415	0.4
1358.5	0.391	0.352	0.413	0.4
1359	0.391	0.352	0.413	0.4
1359.5	0.391	0.352	0.413	0.4
1360	0.391	0.352	0.413	0.4
1360.5	0.391	0.352	0.413	0.4
1361	0.388	0.349	0.413	0.398
1361.5	0.388	0.349	0.413	0.398
1362	0.391	0.352	0.413	0.4
1362.5	0.391	0.352	0.413	0.4
1363	0.391	0.352	0.413	0.4
1363.5	0.391	0.352	0.413	0.4
1364	0.391	0.349	0.413	0.4
1364.5	0.391	0.349	0.413	0.4
1365	0.391	0.352	0.413	0.4
1365.5	0.391	0.352	0.413	0.4
1366	0.391	0.352	0.415	0.398
1366.5	0.391	0.352	0.415	0.398
1367	0.391	0.349	0.413	0.398
1367.5	0.391	0.349	0.413	0.398
1368	0.391	0.349	0.413	0.398
1368.5	0.391	0.349	0.413	0.398
1369	0.391	0.349	0.413	0.4
1369.5	0.391	0.349	0.413	0.4
1370	0.388	0.349	0.413	0.398
1370.5	0.388	0.349	0.413	0.398
1371	0.388	0.349	0.415	0.4
1371.5	0.388	0.349	0.415	0.4
1372	0.388	0.349	0.415	0.4
1372.5	0.388	0.349	0.415	0.4
1373	0.388	0.349	0.413	0.4
1373.5	0.388	0.349	0.413	0.4
1374	0.388	0.349	0.413	0.4
1374.5	0.388	0.349	0.413	0.4

1375	0.388	0.349	0.413	0.4
1375.5	0.388	0.349	0.413	0.4
1376	0.388	0.349	0.413	0.4
1376.5	0.388	0.349	0.413	0.4
1377	0.388	0.349	0.413	0.4
1377.5	0.388	0.349	0.413	0.398
1378	0.388	0.349	0.413	0.398
1378.5	0.388	0.349	0.415	0.4
1379	0.388	0.347	0.415	0.4
1379.5	0.388	0.347	0.413	0.4
1380	0.388	0.347	0.413	0.4
1380.5	0.388	0.347	0.413	0.398
1381	0.388	0.347	0.413	0.398
1381.5	0.388	0.347	0.413	0.398
1382	0.388	0.347	0.413	0.398
1382.5	0.388	0.347	0.413	0.4
1383	0.388	0.347	0.413	0.4
1383.5	0.388	0.347	0.413	0.4
1384	0.388	0.347	0.413	0.4
1384.5	0.388	0.347	0.413	0.398
1385	0.388	0.347	0.413	0.398
1385.5	0.388	0.347	0.413	0.398
1386	0.388	0.347	0.413	0.398
1386.5	0.388	0.347	0.413	0.398
1387	0.388	0.347	0.413	0.398
1387.5	0.388	0.347	0.413	0.398
1388	0.388	0.347	0.413	0.398
1388.5	0.388	0.347	0.413	0.4
1389	0.388	0.347	0.413	0.4
1389.5	0.388	0.347	0.413	0.4
1390	0.388	0.347	0.413	0.4
1390.5	0.388	0.347	0.415	0.4
1391	0.388	0.347	0.415	0.4
1391.5	0.388	0.347	0.415	0.4

1392	0.388	0.347	0.415	0.4
1392.5	0.388	0.347	0.413	0.398
1393	0.388	0.347	0.413	0.398
1393.5	0.388	0.347	0.415	0.4
1394	0.388	0.347	0.415	0.4
